# Kindlin-2 links mechano-environment to proline synthesis and tumor growth

**DOI:** 10.1038/s41467-019-08772-3

**Published:** 2019-02-19

**Authors:** Ling Guo, Chunhong Cui, Kuo Zhang, Jiaxin Wang, Yilin Wang, Yixuan Lu, Ka Chen, Jifan Yuan, Guozhi Xiao, Bin Tang, Ying Sun, Chuanyue Wu

**Affiliations:** 1 Guangdong Provincial Key Laboratory of Cell Microenvironment and Disease Research, Shenzhen Key Laboratory of Cell Microenvironment, Department of Biology and Academy for Advanced Interdisciplinary Studies, Southern University of Science and Technology, Shenzhen, Guangdong 518055, China; 2 Department of Pathology, University of Pittsburgh School of Medicine, Pittsburgh, Pennsylvania 15261 USA; 3 Department of Orthopedic Surgery, Rush University Medical Center, Chicago, Illinois 60612 USA; 4 Department of Biomedical Engineering, Southern University of Science and Technology, Shenzhen, Guangdong 518055, China

## Abstract

Cell metabolism is strongly influenced by mechano-environment. We show here that a fraction of kindlin-2 localizes to mitochondria and interacts with pyrroline-5-carboxylate reductase 1 (PYCR1), a key enzyme for proline synthesis. Extracellular matrix (ECM) stiffening promotes kindlin-2 translocation into mitochondria and its interaction with PYCR1, resulting in elevation of PYCR1 level and consequent increase of proline synthesis and cell proliferation. Depletion of kindlin-2 reduces PYCR1 level, increases reactive oxygen species (ROS) production and apoptosis, and abolishes ECM stiffening-induced increase of proline synthesis and cell proliferation. In vivo, both kindlin-2 and PYCR1 levels are markedly increased in lung adenocarcinoma. Ablation of kindlin-2 in lung adenocarcinoma substantially reduces PYCR1 and proline levels, and diminishes fibrosis in vivo, resulting in marked inhibition of tumor growth and reduction of mortality rate. Our findings reveal a mechanoresponsive kindlin-2-PYCR1 complex that links mechano-environment to proline metabolism and signaling, and suggest a strategy to inhibit tumor growth.

## Abstract (web summary)

The mechano-properties of the Extracellular Matrix (ECM) are important for tumorigenesis. Here, the authors show that the stiffening of the ECM promotes translocation of the focal adhesion protein—Kindlin-2—to the mitochondria, where it interacts with the proline synthesis enzyme PYCR1, stimulating proline synthesis and cell proliferation.

## Introduction

Proline metabolism has important roles in regulation of energy production, protein synthesis, redox balance, and intracellular signaling, in particular under stress or pathological conditions such as cancer^1–5^. Indeed, recent studies have shown that the level of proline is markedly altered in cancer^3,6–8^. PYCR, which is responsible for conversion of Δ^1^‐pyrroline-5-carboxylate into proline, is a key enzyme for proline synthesis. There are three isoforms of PYCR in human, which are encoded by different genes (*PYCR1*, *2*, and *L*). PYCR1 and 2 share a high level (84%) of amino acid sequence similarity and both localize to the mitochondria^1,3,9^. On the other hand, PYCRL is more distinct from PYCR1 and 2 (45% amino acid sequence similarity), and localizes to the cytosol^1,3,9^. It has been shown that PYCR1 is one of the most overexpressed metabolic enzymes in cancer^3,6,7,10–13^. Functional genetic screens have shown that PYCR1 is critical for breast tumor growth^14^ . Indeed, there appears to be an increased demand for proline synthesis in cancer cells^1^. However, despite the compensatory increase of PYCR1 expression, the level of proline is often inadequate for maintaining high level of protein synthesis in proliferating cancer cells^5,6^. This “proline vulnerability” may provide potential therapeutic opportunities for developing novel strategies for cancer treatment. To this end, it is crucial to understand the mechanism through which proline synthesis is regulated.

There is strong interplay between cell metabolism and the microenvironment^2^. For example, in diseases such as cancer, both the mechanical property of cell microenvironment (e.g., extracellular matrix (ECM) stiffness) and cellular metabolism are markedly altered. The alterations of the mechanical property and metabolic activities of cancerous tissues are widely used for diagnosis of cancer^15–17^. In addition, they are attractive targets for cancer therapy. On the one hand, alterations of metabolism can strongly influence the mechanical property of cellular microenvironment such as that of ECM, which in the case of cancer is critical for the growth and dissemination of cancerous cells^18–21^. Many types of human tumors are stiffer than normal tissues, a property has been used to detect tumors^17^. Increased collagen matrix formation and ECM stiffening are key casual factors in tumor development and progression^22–28^. Of note, nearly 25% of amino acids incorporated into collagen are proline. Thus, proline metabolism likely has an important role in collagen synthesis and ECM reprogramming, thereby contributing to tumor development and progression. On the other hand, cellular microenvironment also exerts profound effects on metabolism, as it was recently illustrated in a model of Ras-driven non-small cell lung cancer (NSCLC)^29^. However, how cell mechano-environment has an impact on metabolic activities is not well understood.

Kindlin-2 is a widely expressed and evolutionally conserved protein that is critical for integrin-mediated cell–ECM adhesion and signaling^30–37^. It is well documented that kindlin-2 localizes to focal adhesions^30^, sites where ECM is connected to the actin cytoskeleton^38^. In this study, we show that a fraction of kindlin-2 localizes to the mitochondria and interacts with PYCR1, a key enzyme for proline synthesis. Importantly, kindlin-2 mitochondrion localization and its interaction with PYCR1 are increased in response to ECM stiffening. Concomitantly, the level of PYCR1 and consequently that of proline is increased. Depletion of kindlin-2 markedly reduces the level of PYCR1, increases reactive oxygen species (ROS) production and apoptosis, and abolishes ECM stiffening-induced increase of proline synthesis and cell proliferation. Forced overexpression of PYCR1 reverses to a large extent the inhibition of proline synthesis and cell proliferation induced by the loss of kindlin-2. In vivo, both kindlin-2 and PYCR1 levels are significantly increased in lung adenocarcinoma, which is of greater stiffness compared with that of healthy lung tissues. Using a conditional knockout (KO) strategy, ablation of kindlin-2 from lung adenocarcinoma in mice markedly reduces the levels of PYCR1 and proline, diminished fibrosis, and inhibited tumor growth in vivo, resulting in significant reduction of the mortality rate. We describe below the details of our findings.

## Results

### Identification of PYCR1 and 2 as kindlin-2 binding proteins

**Fig. 1:**
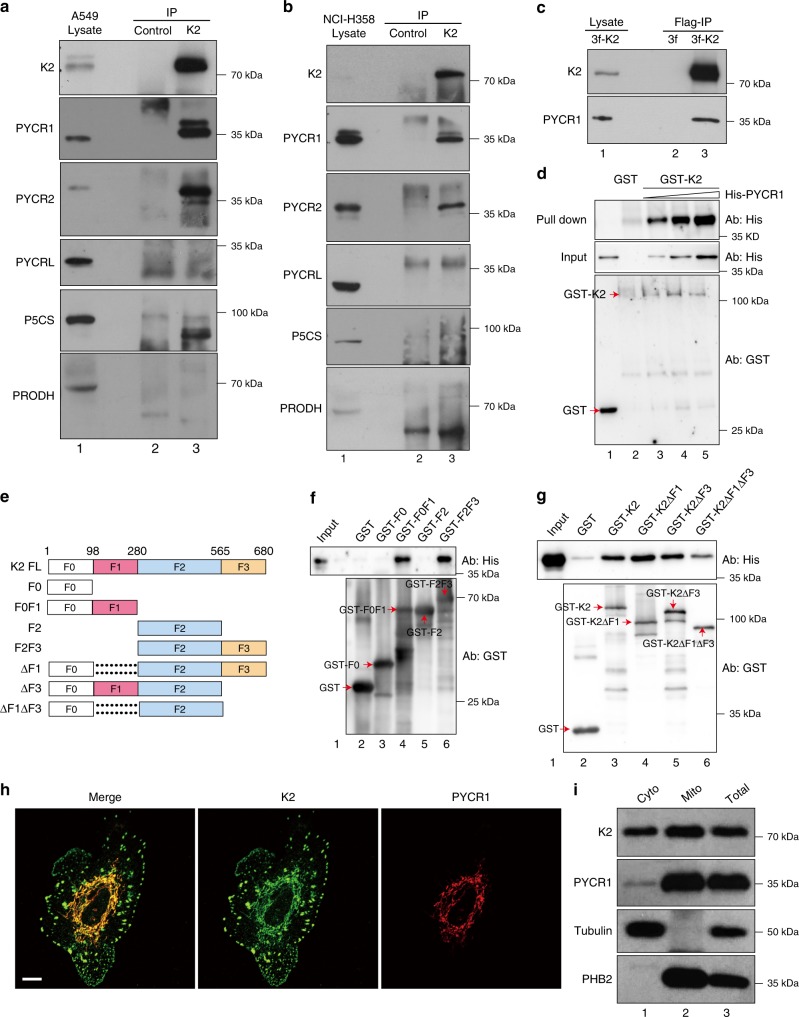
Kindlin-2 interacts with PYCR1 and colocalizes with PYCR1 in mitochondria. **a**, **b** Co-IP of PYCR1 with kindlin-2. Human A549 (**a**) or HCI-H358 (**b**) cells were analyzed by IP with monoclonal anti-kindlin-2 antibody or irrelevant mouse IgG (as a control) as described in the Methods. The cell lysates (lane 1), control IgG (lane 2), and anti-kindlin-2 immunoprecipitates (lane 3) were analyzed by western blotting with antibodies as indicated. **c** Co-IP of PYCR1 with FLAG-kindlin-2. Kindlin-2 KO A549 cells were infected with lentiviral vector encoding 3xFLAG-tagged kindlin-2 (3fl-K2) or control lentiviral vector lacking kindlin-2 sequence (3fl). The 3fl-K2 and control 3fl infectants were analyzed by IP with anti-FLAG antibody. The 3fl-K2 cell lysates (lane 1) and IP samples from 3fl (lane 2) or 3fl-K2 (lane 3) cells were analyzed by western blotting with kindlin-2 or PYCR1 antibodies. **d** GST-kindlin-2 bound to glutathione-Sepharose beads were incubated with increased concentrations (lane 2, 0 μg ml^*−*1^; lane 3, 10 μg ml^*−*1^; lane 4, 20 μg ml^*−*1^; lane 5, 40 μg ml^*−*1^) of purified His-tagged PYCR1. GST-kindlin-2 fusion protein pulldown was analyzed as described in the Methods. The sample in lane 1 was prepared as that in lane 4, except GST-kindlin-2 was replaced with GST. **e**–**g** GST-tagged full-length or mutant forms of kindlin-2 (illustrated in **e**) or GST alone (as a negative control) were used to pull down His-tagged PYCR1 as described in the Methods. The inputs, GST and GST-kindlin-2 fusion protein pulldowns, were analyzed by western blotting with antibodies against His and GST, respectively. **h** A549 cells were plated on fibronectin-coated coverslips and dually stained with mouse monoclonal anti-kindlin-2 and rabbit polyclonal anti-PYCR1 antibodies. The primary antibodies were detected with Alexa Fluor 488-conjugated anti-mouse IgG or Alexa Fluor 647-conjugated anti-rabbit IgG secondary antibodies. Scale bar = 10 μm. **i** The cytosolic fraction (Cyto, lane 1), mitochondrial fraction (Mito, lane 2), and total cell lysates (Total, lane 3) were analyzed by western blotting with antibodies to kindlin-2, PYCR1, tubulin, and prohibitin-2 (PHB2) as indicated

We identified PYCR1 and its closely related isoform PYCR2 as kindlin-2-binding proteins using an anti-kindlin-2 immunoprecipitation (IP)/Nanoscale liquid chromatography coupled to tandem mass spectrometry (nano LC-MS/MS) approach. Human A549 NSCLC cells were used in these experiments (see Methods for detail). PYCRL and other proline-metabolic enzymes such as pyrroline-5-carboxylate synthase (P5CS) and proline dehydrogenase (PRODH) were not detected in the same anti-kindlin-2 IP/nano LC-MS/MS experiment. To confirm these results, we analyzed the anti-kindlin-2 IP samples by western blotting with antibodies to PYCR1, 2, L, P5CS, and PRODH, respectively. Consistent with the nano LC-MS/MS results, PYCR1 and 2 were readily detected in anti-kindlin-2 (Fig. 1a, lane 3) but not in control (Fig. 1a, lane 2) IPs. By contrast, no PYCRL, P5CS, and PRODH were specifically co-IPed with kindlin-2 (Fig. 1a, lane 3). We repeated the IP/western blotting analyses using another NSCLC cell line (NCI-H358) and obtained similar results (Fig. 1b). As PYCR1 is critically involved in the tumorigenesis^3,6,7,10–14^, we sought to further characterize the interaction of kindlin-2 with PYCR1. To do this, we generated kindlin-2 KO A549 cells using the CRISPR/Cas9 system and infected them with lentiviral vector encoding 3xFLAG-kindlin-2 (3fl-K2) or control lentiviral vector lacking kindlin-2 sequence (3fl). The 3fl-K2 and control 3fl cells were analyzed by IP with anti-FLAG antibody and western blotting. The results showed that PYCR1 was co-immunoprecipitated with 3xFLAG-kindlin-2 from 3fl-K2 (Fig. 1c, lane 3) but not control 3fl (Fig. 1c, lane 2) cells. Next, we generated and purified recombinant glutathione *S*-transferase (GST)-tagged kindlin-2 and His-tagged PYCR1, respectively, and tested the binding in a GST pulldown assay. Recombinant His-PYCR1 was readily pulled down by purified GST-kindlin-2 (Fig. 1d, lane 3) but not by GST alone (Fig. 1d, lane 2). Furthermore, the amount of His-PYCR1 pulled down by GST-kindlin-2 was increased when the latter was incubated with higher concentrations of His-PYCR1 (Fig. 1d, lanes 3–5). These results suggest that kindlin-2 binds PYCR1 directly. To map the kindlin-2 domains that mediate the binding, we generated GST-fusion proteins containing various kindlin-2 subdomains (Fig. 1e) and tested their PYCR1-binding activities. The results showed that kindlin-2 F0F1 (Fig. 1f, lane 4) or F2F3 (Fig. 1f, lane 6), but not F0 (Fig. 1f, lane 3) or F2 (Fig. 1f, lane 5), interacted with PYCR1. Consistent with these results, deletion of both F1 and F3 (Fig. 1g, lane 6), but not that of either F1 (Fig. 1g, lane 4) or F3 (Fig. 1g, lane 5), significantly reduced PYCR1 binding, suggesting that the PYCR1 binding is mediated primarily by kindlin-2 F1 and F3 subdomains.

### Kindlin-2 colocalizes with and binds PYCR1 in mitochondria

**Fig. 2:**
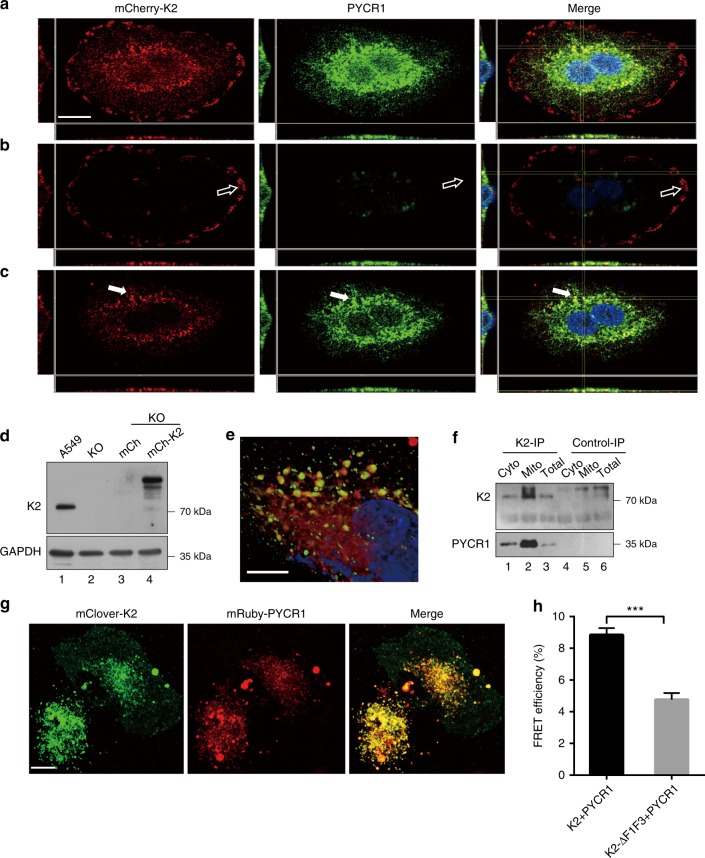
Formation of the kindlin-2-PYCR1 complex in cells. **a**–**e** Kindlin-2 KO A549 cells were infected with lentiviral vector encoding mcherry-tag kindlin-2 (mCh-K2) or mcherry vector lacking kindlin-2 sequence (mCh). Three days later, A549 cells (lane 1), kindlin-2 KO cells (lane 2), mCh infectants (lane 3), and mCh-K2 infectants (lane 4) were analyzed by western blotting with antibodies recognizing kindlin-2 and GAPDH (**d**). Kindlin-2 KO cells expressing mcherry-kindin-2 were stained with anti-PYCR1 antibody and DAPI, and analyzed by confocal microscopy. The maximum-intensity projection views of a representative A549 mCh-K2 cell reconstructed from Z-series images taken at various heights (**a**). Extended focal planes of focal adhesions show that mcherry-kindlin-2 is concentrated in focal adhesions, whereas there is virtually no PYCR1 in focal adhesions (open arrows) (**b**). Image layers taken above focal adhesions show that a fraction of mcherry-kindlin-2 is colocalized with PYCR1 in the mitochondria (solid arrows) (**c**). Fluorescence images of mcherry-kindlin-2 (red), PYCR1 (green), and merged images with DAPI (blue) are shown in the left, middle, and right column, respectively. Scale bar = 15 μm. High-magnification images of mcherry-kindlin-2 (red), PYCR1 (green), and DAPI (blue) taken with focal plane above focal adhesions (**e**). Scale bar = 7.5 μm. **f** Co-IP of PYCR1 with kindlin-2 from mitochondrial fraction. Cytosolic fraction (lane 1), mitochondrial fraction (lane 2), and total lysates (lane 3) from A549 cells were analyzed by IP with anti-kindlin-2 antibody (lanes 1–3) or irrelevant mouse IgG (lanes 4–6). The samples were analyzed by western blotting with antibodies to kindlin-2 and PYCR1. **g**, **h** FLIM-FRET analyses. Kindlin-2 KO cells were transfected with vectors encoding mClover-kindin-2 or mClover-F1 and F3 deletion mutant of kindlin-2 and mRuby-PYCR1, and analyzed by FLIM. The fluorescence images of mClover-kindin-2 (green), mRuby-PYCR1 (red), and merged images are shown (**g**). Scale bar = 10 μm. The efficiencies of FRET between the pair of mClover-kindlin-2 and mRuby-PYCR1, or that of mClover-F1 and F3 deletion mutant of kindlin-2 and mRuby-PYCR1 were analyzed (**h**, at least *n* = 15 cells per group were counted from at least three independent experiments). Data are shown as mean ± SEM using two-tailed unpaired Student’s *t*-test, ***p < 0.001

We next analyzed the subcellular localization of the kindlin-2-PYCR1 complex. Immunofluorescent staining showed that, as expected, PYCR1 was localized primarily in the mitochondria (Supplementary Fig. 1). Double staining of the cells with mouse monoclonal anti-kindlin-2 and rabbit polyclonal anti-PYCR1 antibodies showed that a fraction of kindlin-2 was colocalized with PYCR1 in the mitochondria (Fig. 1h). PYCR1, however, was not detected in focal adhesions where kindlin-2 was also present (Fig. 1h). These results suggest that the kindlin-2-PYCR1 complex is localized primarily in mitochondria. Next, we isolated mitochondrial and non-mitochondrion cytosolic fractions from the cells (see Methods). To confirm that these fractions are devoid of cross-contamination, we probed them as well as total cell lysates (as a positive control) with antibodies recognizing prohibitin-2 (PHB2), a mitochondrial protein, and tubulin, a cytosolic protein, respectively. As expected, PHB2 was detected in the mitochondrial fraction (Fig. 1i, lane 2) and total cell lysates (Fig. 1i, lane 3) but not in the cytosolic fraction (Fig. 1i, lane 1). Conversely, tubulin was detected in the cytosolic fraction (Fig. 1i, lane 1) and total cell lysates (Fig. 1i, lane 3) but not in the mitochondrial fraction (Fig. 1i, lane 2). Next, we probed the fractions with anti-kindlin-2 and anti-PYCR1 antibodies. The majority of PYCR1 was detected in the mitochondrial fraction (Fig. 1i, lane 2), albeit a small amount of PYCR1 was also detected in the cytosolic fraction (Fig. 1i, lane 1). Notably, abundant kindlin-2 was detected in both the mitochondrial (Fig. 1i, lane 2) and cytosolic (Fig. 1i, lane 1) fractions. The results of these biochemical analyses are highly consistent with those of immunofluorescence staining, suggesting that a fraction of kindlin-2 is colocalized with PYCR1 in the mitochondria. To further test this, we expressed mCherry-tagged kindlin-2 in kindlin-2 KO cells, which was confirmed by western blotting (Fig. 2d), and stained the cells with anti-PYCR1 antibody and analyzed them by confocal microscopy (Fig. 2a–e). Similar to the results obtained with anti-kindlin-2 antibody, mCherry-kindlin-2 was colocalized with PYCR1 in the mitochondria (Fig. 2a–e). Again, no PYCR1 was detected in focal adhesions where kindlin-2 was also present (Fig. 2a, b). Collectively, these results confirm that a fraction of kindlin-2 is colocalized with PYCR1 in the mitochondria.

To further test the formation of the kindlin-2-PYCR1 complex in cells, we immunoprecipitated kindlin-2 from the mitochondrion, cytosol, and total cell (as a positive control) lysates, respectively, and analyzed the samples by western blotting. The results showed that PYCR1 was co-immunoprecipitated with kindlin-2 from all three lysates, with the largest amount of PYCR1 found in anti-kindlin-2 immunprecipitates from the mitochondrial fraction (Fig. 2f, compare lane 2 with lanes 1 and 3). No PYCR1 was found in control IgG immunprecipitates (Fig. 2f, lanes 4–6). Collectively, these studies suggest that the majority of the kindlin-2-PYCR1 complex is localized in the mitochondria, albeit a small amount of the kindlin-2-PYCR1 complex may also be present in subcellular compartments other than in the mitochondria.

To confirm that kindlin-2 and PYCR1 form a complex in cells, we performed fluorescence resonance energy transfer (FRET) analyses. To do this, we co-expressed mRuby-tagged PYCR1 and mClover-tagged wild type (Fig. 2g) or F1F3-deletion mutant form of kindlin-2 in cells. Significant FRET signals were detected between mClover-kindlin-2 and mRuby-PYCR1 (Fig. 2h), indicating that kindlin-2 and PYCR1 indeed form a complex in cells. Consistent with the results of the GST-pulldown experiments (Fig. 1e–g), deletion of F1 and F3 domains significantly reduced FRET signals (Fig. 2h).

### Depletion of kindlin-2 reduces PYCR1 and proline levels

**Fig. 3:**
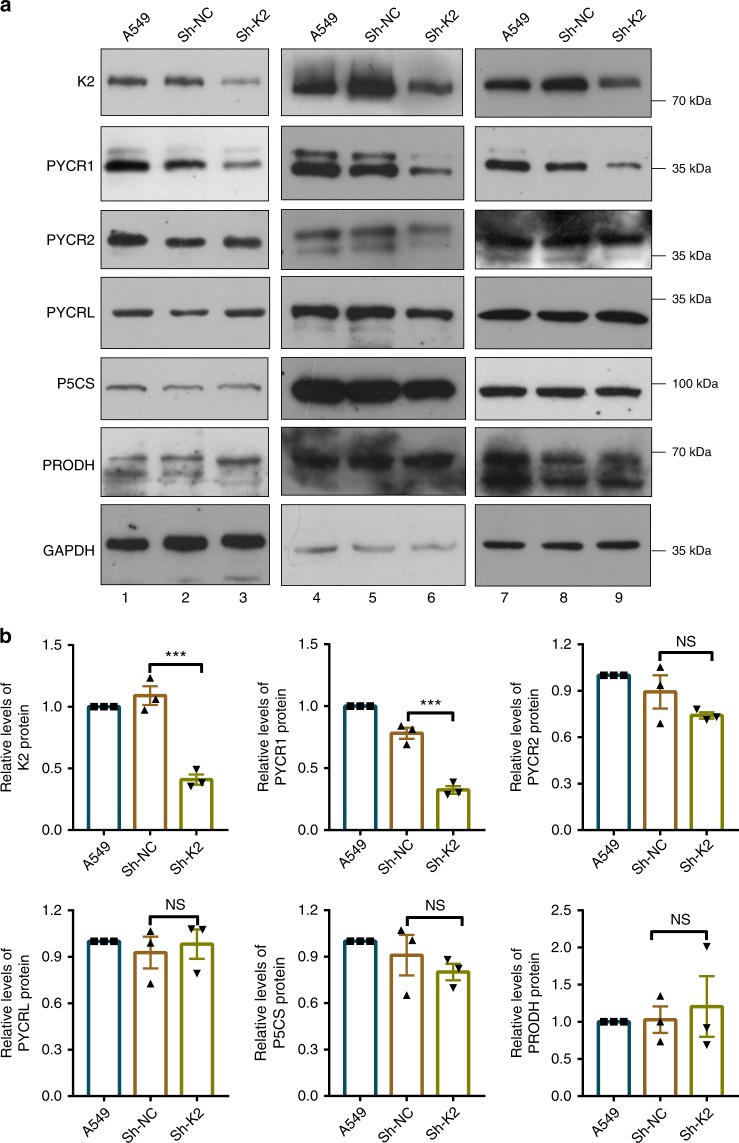
The effects of kindlin-2 depletion on PYCR, P5CS, and PRODH levels. **a** A549 cells were infected with control (Sh-NC) or kindlin-2 shRNA (Sh-K2) lentivirus for 5 days and then analyzed by western blot with antibodies recognizing kindlin-2, PYCR1, PYCR2, PYCRL, P5CS, PRODH, and GAPDH (as a loading control). Three independent experiments were shown. **b** Protein levels of kindlin-2, PYCR1, PYCR2, PYCRL, P5CS, and PRODH relative to that of GAPDH in Sh-NC and Sh-K2 cells were quantified by densitometric analysis of western blottings and compared with those in the A549 cells (normalized to 1, *n* = 3). Data represent mean ± SEM from three independent experiments using one-way ANOVA with Tukey–Kramer post-hoc analyses, ***p < 0.001, NS, no significance

**Fig. 4:**
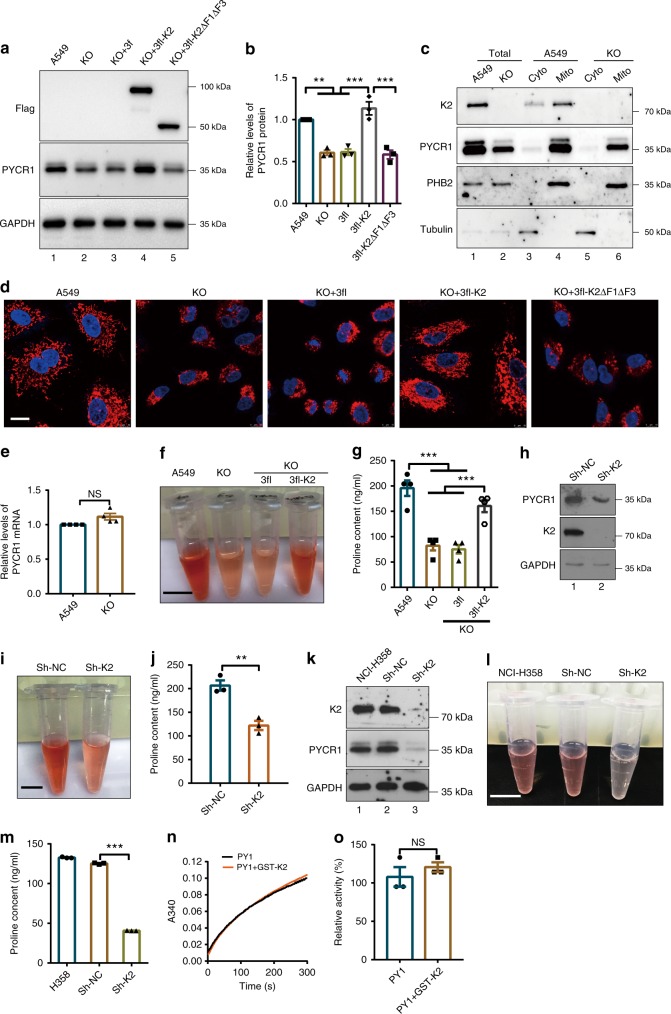
Kindlin-2 regulates PYCR1 and proline levels. **a** Kindlin-2 KO cells were infected with lentiviral vectors encoding 3xFLAG-tagged kindlin-2 (3fl-K2), 3xFLAG-tagged kindlin-2 F1, and F3 deletion mutant (3fl-K2∆F1∆F3) or 3xFLAG vector lacking kindlin-2 sequence (3fl). Three days later, the cells were analyzed by western blotting. **b **Protein levels of PYCR1 relative to that of GAPDH were quantified by densitometric analyses. The levels of PYCR1 in KO, 3fl, 3fl-K2, or 3fl-K2∆F1∆F3 cells were compared with that of A549 cells (normalized to 1) (*n* = 3). **c** The cytosolic (lane 3 and 5), mitochondrial (lane 4 and 6), and total (lane 1 and 2) lysates from A549 or kindlin-2 KO cells were analyzed by western blotting. **d** The cells were immunofluorescently stained with DAPI and anti-PYCR1 antibodies. Scale bar = 10 μm. **e** The mRNA levels of PYCR1 in kindlin-2 KO cells were analyzed by RT-PCR (*n* = 4) and compared with those in A549 cells (normalized to 1). **f**, **g** The proline levels were analyzed. The results were visualized (**f**) and quantified (**g**, *n* = 4). Scale bar = 1 cm. **h**–**j** A549 cells infected with kindlin-2 shRNA (Sh-K2) or control (Sh-NC) lentivirus were analyzed by western blotting (**h**). The proline levels were analyzed. The results were visualized (**i**) and quantified (**j**, *n* = 3). Scale bar = 1cm. **k**–**m** NCI-H358 cells were infected with kindlin-2 shRNA (Sh-K2) or control (Sh-NC) lentivirus for 5 days and then analyzed by western blotting (**k**). **l**, **m** The proline levels were analyzed. The results were visualized (**l**) and quantified (**m**, *n* = 3). Scale bar = 1 cm. **n**, **o** The effect of kindlin-2 on PYCR1 enzyme activity. The enzyme activity of purified PYCR1 (PY1) with or without GST-kindlin-2 (GST-K2) was analyzed. **n** The absorbance at 340 nm during the first 5 min of the reaction. **o** The relative activities of PYCR1 with or without GST-Kindlin-2 were calculated from **n** (*n* = 3). Data in **b**, **e**, **g**, **j**, **m**, and **o** represent means ± SEM. Statistical significance was calculated using one-way ANOVA with Tukey–Kramer post-hoc analysis in **b**, **j**, and **m** or using two-tailed unpaired Student’s *t*-test in **e**, **j**, and **o**, **P < 0.01; ***p < 0.001. NS, no significance

We next sought to determine the function of kindlin-2 in regulation of PYCR1. To do this, we knocked down kindlin-2 from A459 cells and compared the level of PYCR1 in kindlin-2 knockdown and control cells. The results showed that the level of PYCR1 was significantly reduced in response to knockdown of kindlin-2 (Fig. 3a, compare lanes 2 and 3, 5 and 6, 8 and 9; Fig. 3b). By contrast, knockdown of kindlin-2 did not significantly reduce the levels of PYCR2, PYCRL, P5CS, and PRODH (Fig. 3a, compare lanes 2 and 3, 5 and 6, 8 and 9; Fig. 3b). To further investigate this, we analyzed the messenger RNA and protein levels of PYCR1 in response to loss of kindlin-2. The results showed that loss of kindlin-2 significantly reduced the protein (Fig. 4a, compare lanes 1 and 2; Fig. 4b–d) but not mRNA (Fig. 4e) level of PYCR1. Although the level of PYCR1 was significantly reduced in response to loss of kindlin-2 (Fig. 4a, compare lanes 1 and 2), the majority of PYCR1 in kindlin-2 KO cells, similar to that in control cells, remained to localize in the mitochondria (Fig. 4c, compare lanes 4 and 6 with 3 and 5), suggesting that loss of kindlin-2 did not significantly change the compartmentation of PYCR1. To confirm that the reduction of the PYCR1 protein level is caused by impairment of the interaction with kindlin-2, we re-expressed wild-type kindlin-2 and the PYCR1-binding defective ΔF1ΔF3 mutant (Fig. 1e), respectively, in kindlin-2 KO cells (Fig. 4a). Whereas expression of wild-type kindlin-2 in kindlin-2 KO cells effectively restored the level of PYCR1 (Fig. 4a, lane 4; Fig. 4b–d), expression of the PYCR1-binding defective mutant of kindlin-2 failed to do so (Fig. 4a, lane 5; Fig. 4b–d), suggesting that the interaction with kindlin-2 is critical for control of the protein level of PYCR1. Although the level of PYCR1 is significantly reduced in response to depletion of kindlin-2, no significant changes of ubiquitination, tyrosine phosphorylation, or serine phosphorylation of PYCR1 were observed under the same condition (Supplementary Fig. 2A). Consistent with the lack of PYCR1 ubiquitination in response to depletion of kindlin-2 (Supplementary Fig. 2A), treatment with MG-132 failed to reverse the reduction of PYCR1 level induced by the knockdown of kindlin-2 (Supplementary Fig. 2B, compare lanes 3 and 4; Supplementary Fig. 2C). Similar results were obtained with kindlin-2 KO cells (Supplementary Fig. 2D, compare lanes 2 and 3). Interestingly, treatment of kindlin-2 KO or knockdown cells with leupeptin partially reversed the reduction of PYCR1 level induced by the loss of kindlin-2 (Supplementary Fig. 2B, compare lanes 3 and 5; Supplementary Fig. 2C; and Supplementary Fig. 2D, compare lanes 2 and 4), suggesting that a leupeptin-sensitive protease is likely involved, either directly or indirectly, in degradation of PYCR1 in response to loss of kindlin-2.

As PYCR1 is a key enzyme for proline synthesis, we reasoned that reduction of the PYCR1 level might have an impact on the cellular level of proline. To test this, we compared proline levels in kindlin-2 KO and control A549 cells. As expected, KO of kindlin-2 indeed significantly reduced the level of proline (Fig. 4f, g). Similarly, knockdown of kindlin-2 from A549 cells (Fig. 4h– j) or NCI-H358 cells (Fig. 4k–m) by RNA interference also significantly reduced the levels of PYCR1 (Fig. 4h–k) and proline level (Fig. 4i–m). Re-expression of kindlin-2 in kindlin-2 KO cells nearly completely restored the proline level (Fig. 4f, g). Biochemical analyses of the enzyme activity of PYCR1 showed that it was not altered in the presence or absence of kindlin-2 (Fig. 4n, o). Collectively, these results suggest that kindlin-2 is a key regulator of the PYCR1 level and consequently the proline level in cells.

### Kindlin-2 regulates cell survival and proliferation

**Fig. 5:**
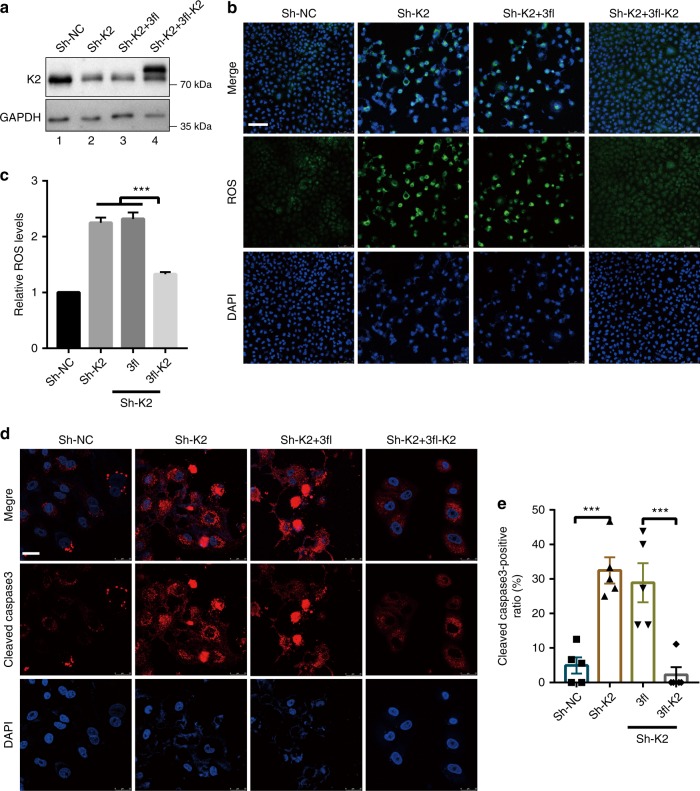
Depletion of kindlin-2 increases ROS production and apoptosis. **a**–**c** A549 cells were infected with K2 shRNA (Sh-K2) lentivirus or control lentivirus (Sh-NC). Two days after the infection, the cells were infected with lentiviral expression vectors encoding 3xFLAG-tagged kindlin-2 (3fl-K2) or 3xFLAG empty vector (3fl). Three days after the lentiviral infection, the cells were analyzed by western blotting with antibodies recognizing K2 or GAPDH (as a loading control) (**a**). The levels of ROS were analyzed using DHE fluorescence probe as described in the Methods (**b**). Scale bar, 75 μm. The mean fluorescence intensities (MFI) were calculated using the Image-pro plus software from two different experiments (**c**, at least *n* = 15 field per group were counted from at least three independent experiments). **d** The cells (as specified in the figure) were immunofluorescently stained with DAPI and anti-cleaved caspase-3 antibody. Scale bar = 25 μm. The percentages of cleaved caspase-3-positive cells were quantified (**e**) as described in the Methods. Replicates (*n* = 5) from one representative experiment are shown. Data in **c** and **e** are presented as mean ± SEM using one-way ANOVA with Tukey–Kramer post-hoc analysis, *P < 0.05; ***p < 0.001

**Fig. 6:**
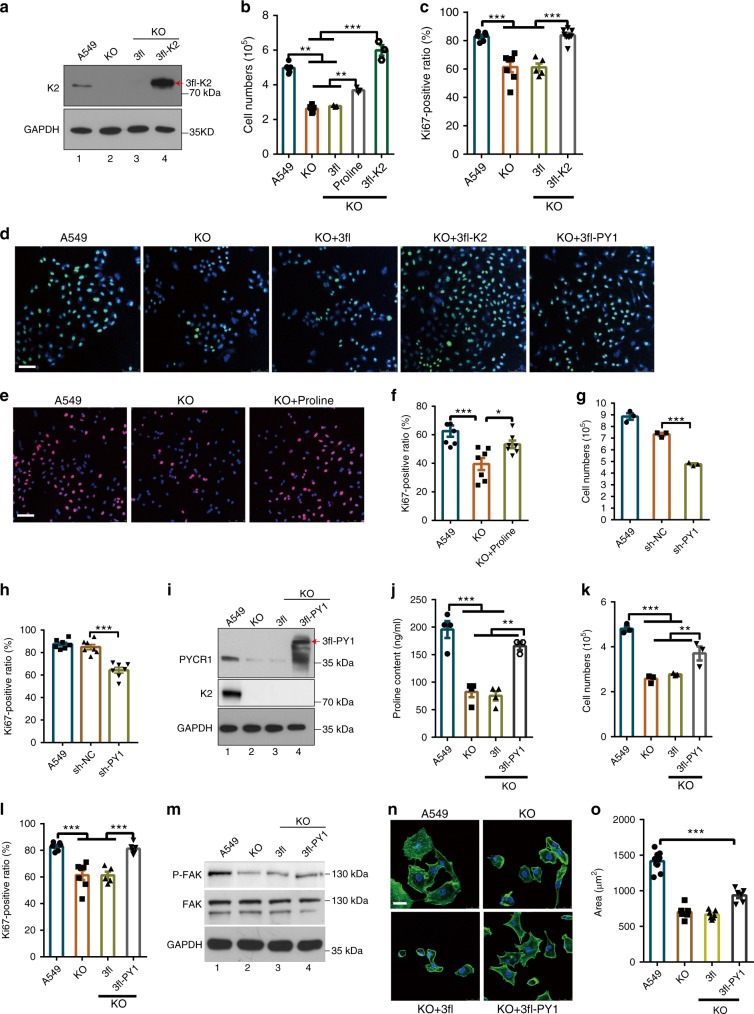
Kindlin-2 regulates cell proliferation through PYCR1. **a**–**d** Kindlin-2 KO A549 cells were infected with lentiviral vectors encoding 3xFLAG-tagged kindlin-2 (3fl-K2) or 3xFLAG empty vector (3fl). Three days later, the cells were analyzed by western blotting (**a**). The cells were seeded in six-well plate (1 × 10^5^/well), cultured for 2 days, and the number of cells was counted (**b**, *n* = 3). Cells were stained with DAPI and anti-Ki67 antibody (**d**). Scale bar, 75 μm. The percentages of Ki67-positive cells were quantified (**c**, *n* = 5). **e**, **f** Kindlin-2 KO A549 cells were treated with proline for 3 days (interval of treatment = 12 h). Cells were stained with DAPI and anti-Ki67 antibody (**e**). Scale bar, 75 μm. The percentages of Ki67-positive cells were quantified (**f**, *n* = 5). **g, h **A549 cells were infected with PYCR1 shRNA (Sh-PY1) or control (Sh-NC) lentivirus for 5 days. The cells were seeded in six-well plate (1×10^5^/well), cultured for 2 days, and the numbers of cells were counted (**g**, *n* = 3). Cells were stained with DAPI and anti-Ki67 antibody and the percentages of Ki67-positive cells were quantified (**h**, *n* = 5). **i**–**l** Kindlin-2 KO A549 cell lines (ko) were infected with lentiviral vectors encoding 3×FLAG tagged PYCR1 (3fl-PY1) or 3xFLAG empty vector (3fl). Three days later, the cells were analyzed by western blotting (**i**). The proline levels in KO, KO+3fl, and KO+3fl-PY1 cells were quantified and compared with that of A549 (**j**, *n* = 3). The cells were seeded in growth medium in six-well plate (1×10^5^/well), cultured for 2 days, and then the numbers of cells were counted (**k**, *n* = 3). Cells were stained with DAPI and anti-Ki67 antibody. The percentages of Ki67-positive cells were quantified (**l**, *n* = 5). **m**–**o** Kindlin-2 KO A549 cells were infected with lentiviral vectors encoding 3xFLAG-tagged PYCR1 (3fl-PY1) or 3xFLAG vector (3fl). Three days later, the cells were analyzed by western blotting (**m**). Cells were immunofluorescently stained with Alexa Fluor-conjugated phalloidin (**n**). Scale bar, 25 μm. Cell areas (**o**) were quantified using Image J. At least 50 cells from each group were analyzed (*n* = 5). Data in **b**, **c**, **f**, **g**, **h**, **j**–**l**, and **o** are presented as mean ± SEM using one-way ANOVA with Tukey–Kramer post-hoc analysis, *P < 0.05; **P < 0.01; ***p < 0.001

Concomitant to the reduction of PYCR1 and proline levels, knockdown of kindlin-2 (Fig. 5a, lane 2) significantly increased ROS production (Fig. 5b, c) and apoptosis (Fig. 5d, e). Re-expression of FLAG-kindlin-2 in kindlin-2 knockdown cells (Fig. 5a, lane 4) effectively reversed the increases of ROS production (Fig. 5b, c) and apoptosis (Fig. 5d, e). Similarly, loss of kindlin-2 reduced the cell number and the percentage of Ki67-positive cells (Fig. 6b–d). Again, re-expression of FLAG-kindlin-2 in kindlin-2 KO cells (Fig. 6a, lane 4) effectively reversed the reduction of the cell number (Fig. 6b) and proliferation (Fig. 6c, d). These results suggest a crucial role of kindlin-2 in regulation of ROS production, cell survival, and proliferation. Addition of proline to kindlin-2 KO cells partially reversed the inhibition of cell proliferation (Fig. 6e, f), suggesting that the reduction of the proline level induced by the loss of kindlin-2 is responsible for, at least in part, suppression of cell proliferation.

### Kindlin-2 regulates proline synthesis through PYCR1

We next tested whether the reduction of the proline level and proliferation of kindlin-2 KO cells was caused by reduction of PYCR1 level. Consistent with previous studies^6,13,39^, knockdown of PYCR1 significantly reduced the cell number (Fig. 6g) and the percentage of Ki67-positive cells (Fig. 6h and Supplementary Fig. 3). Next, we infected kindlin-2 KO cells with a lentiviral vector encoding 3xFLAG-PYCR1 (3fl-PY1) or a control lentiviral vector lacking PYCR1 sequence (3fl). Expression of FLAG-PYCR1 in 3xFLAG-PYCR1 (Fig. 6i, lane 4) but not control (Fig. 6i, lane 3) infectants was confirmed by western blotting. Importantly, expression of 3xFLAG-PYCR1 in kindlin-2 KO cells restored to a large extent the proline level (Fig. 6j), cell number (Fig. 6k), and the percentage of Ki67-positive cells (Fig. 6d, l), suggesting that kindlin-2 regulates the proline level and cell proliferation through, at least in part, control of PYCR1 level. As expected, loss of kindlin-2 also impaired Tyr397 phosphorylation of focal adhesion kinase (FAK) (Fig. 6m, compare lane 2 with lane 1), an integrin-proximal signaling event, and cell spreading (Fig. 6n, o). However, expression of 3xFLAG-PYCR1 in kindlin-2 KO cells did not rescue the defect on FAK Tyr397 phosphorylation (Fig. 6m, compare lanes 3 and 4) and reversed only modestly (~28%) the defect on cell spreading (Fig. 6n, o). These results suggest that the kindlin-2-PYCR1 signaling axis has a major impact on kindlin-2-dependent regulation of cell proliferation, albeit it may not have an essential role in integrin-proximal signaling events such as FAK Tyr397 phosphorylation and may have only a modest role in cytoskeletal remodeling.

### Mechano-regulation of kindlin-2-PYCR1 interaction

**Fig. 7:**
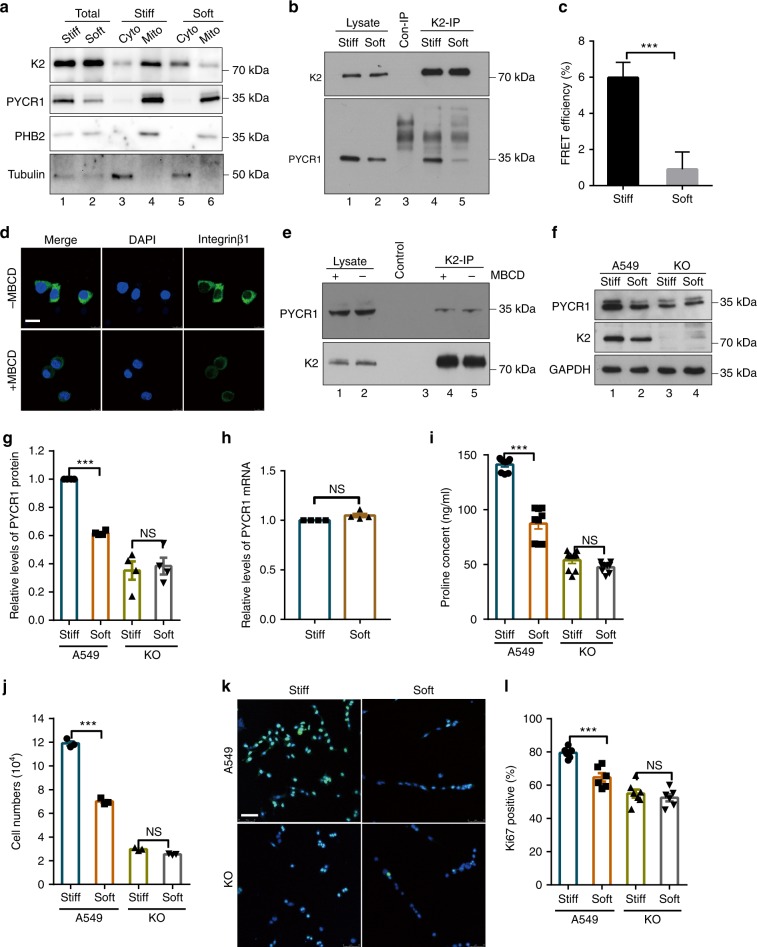
ECM stiffening regulates kindlin-2-PYCR1 interaction. **a** The cytosolic (lanes 3 and 5), mitochondrial (lanes 4 and 6), and total (lanes 1 and 2) lysates from A549 cells on stiff (elastic modulus of 40 kPa) or soft (elastic modulus of 0.35 kPa) hydrogels were analyzed by western blotting. **b** A549 cells plated on stiff or soft hydrogels for 48 h were analyzed by IP and western blotting. Lane 3, the sample was prepared as that of lane 4, except anti-kindlin-2 antibody was substituted with irrelevant mouse IgG. **c** A549 cells expressing mClover-kindin-2 and mRuby-PYCR1 on stiff or soft hydrogels were analyzed by FLIM-FRET (> 15 cells per group were counted from at least three independent experiments). **d**, **e** A549 cells on soft hydrogels were pretreated with or without 10 mM MBCD for 1 h and stained with anti-β1 integrin antibody (**d**) or analyzed by IP with anti-kindlin-2 antibody (**e**). Scale bar in **d**, 25 μm. The lysates (lanes 1 and 2), control IgG (lane 3), and anti-kindlin-2 immunoprecipitates (lanes 4 and 5) were analyzed by western blotting (**e**). **f**–**l** A549 or kindlin-2 KO cells on stiff or soft hydrogels were analyzed by western blotting (**f**). Protein levels of PYCR1 relative to that of GAPDH were quantified by densitometric analyses and compared with that of A549 cells (normalized to 1) (**g**, *n* = 3). The mRNA level of PYCR1 in A549 cells on soft hydrogels was analyzed by RT-PCR and compared with that in A549 cells on stiff hydrogels (normalized to 1) (**h**, *n* = 3). The proline levels in A549 or kindlin-2 KO cells on stiff or soft hydrogels were analyzed (**i**, *n* = 3). The cells (3.5 × 10^4^/gel) were plated on stiff or soft hydrogels, cultured for 2 days, the number was counted (**j**, *n* = 3) and cells were stained with DAPI and anti-Ki67 antibody (**k**). Scale bar, 75 μm. The percentages of Ki67-positive cells were quantified (**l**, *n* = 5). Data in **c**, **g**, **h**, **i**, **j**, and **l** represent means ± SEM. Statistical significance was calculated using one-way ANOVA with Tukey–Kramer post-hoc analysis (**g**, **i**, **j**, **l**) or two-tailed unpaired Student’s *t*-test (**c**, **h**). ***p < 0.001. NS, no significance

We next sought to test whether kindlin-2 and PYCR1 localization and their complex formation were influenced by mechano-environment. To do this, we plated the cells on ECM with different stiffness^37,40–42^ and analyzed the effects on kindlin-2 and PYCR1 localization and their complex formation. The results showed that PYCR1 was localized primarily in the mitochondria in cells irrespective of ECM stiffness (Fig. 7a, compare lane 4 with lane 6 and lane 3 with lane 5). By marked contrast, ECM stiffening significantly increased the amount of kindlin-2 in the mitochondria and concomitantly reduced the amount of kindlin-2 in the cytosol (Fig. 7a, compare lane 4 with lane 6 and lane 3 with lane 5). Furthermore, co-IP experiments showed that the amount of PYCR1 complexed with kindlin-2 was significantly increased in response to ECM stiffening (Fig. 7b, compare lanes 4 and 5). To confirm that ECM stiffening promotes the kindlin-2-PYCR1 complex formation, we performed FRET analyses by plating cells expressing mClover-kindin-2 and mRuby-PYCR1 on ECM with different stiffness. Consistent with the results obtained by co-IP (Fig. 7b), the FRET signal between mClover-kindin-2 and mRuby-PYCR1 was significantly increased in response to ECM stiffening (Fig. 7c). Collectively, these results suggest that kindlin-2 localization to the mitochondria and its complex formation with PYCR1 are regulated by mechano-environment: they are enhanced when cells are in contact with stiff ECM and reduced when cells are in contact with soft ECM. Previous studies have shown that soft ECM promotes integrin internalization^42^. To test whether integrin internalization is involved in soft ECM-induced downregulation of the kindlin-2-PYCR1 complex, we treated the cells that were plated on soft ECM with methyl-β-cyclodextrin (MBCD), a caveolae/raft inhibitor that is known to suppress soft ECM-induced integrin internalization^42^. Consistent with the previous studies^42^, treatment of the cells with MBCD inhibited integrin internalization (Fig. 7d). However, inhibition of integrin internalization did not alter the formation of the kindlin-2–PYCR1 complex (Fig. 7e, compare lanes 4 and 5), suggesting that soft ECM-induced inhibition of the kindlin-2–PYCR1 complex is independent of integrin internalization. As expected, kindlin-2 and PYCR1 localization and proline synthesis analyses showed that they were not altered in the presence or absence of MBCD on soft substrates (Supplementary Fig. 4).

Consistent with a positive role of the kindlin-2-PYCR1 complex formation in regulation of the PYCR1 level, the protein (Fig. 7f, compare lanes 1 and 2; Fig. 7g) but not mRNA (Fig. 7h) level of PYCR1 was increased in response to ECM stiffening. Of note, the ECM stiffening-induced increase of PYCR1 protein level was abolished in the absence of kindlin-2 (Fig. 7f, compare lanes 3 and 4; Fig. 7g). Similarly, both the proline level and cell proliferation were increased in response to ECM stiffening (Fig. 7i–l). Again, the ECM stiffening-induced increases of the proline level and cell proliferation were abolished in the absence of kindlin-2 (Fig. 7i–l). Collectively, these results demonstrate that kindlin-2 is essential for ECM stiffening-induced upregulation of PYCR1 and proline levels, and cell proliferation.

### Kindlin-2 and PYCR1 expression in lung adenocarcinoma

**Fig. 8:**
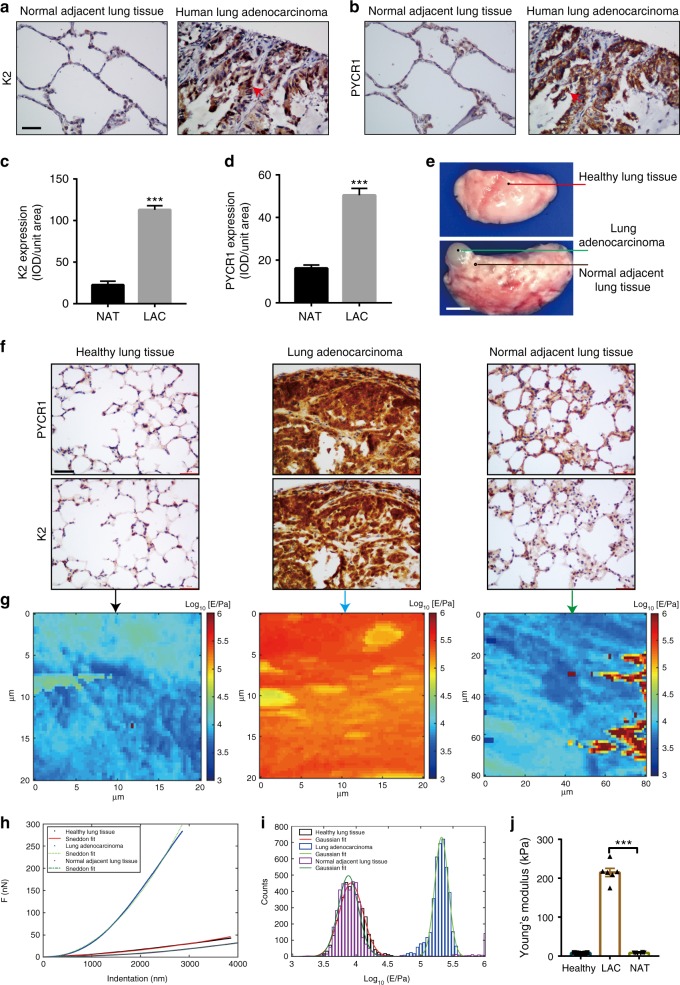
Elevated levels of kindlin-2, PYCR1, and tissue stiffness in lung adenocarcinoma. **a**–**d** Human lung adenocarcinoma (LAC) and normal adjacent lung tissues (NAT) were stained with kindlin-2 (**a**) or PYCR1 (**b**) antibodies as indicated. Red arrows indicate tumor regions. Scale bar, 20 μm. Kindlin-2 (**c**) and PYCR1 (**d**) immunohistochemical (IHC) staining of human lung adenocarcinoma and normal adjacent lung tissues was quantified as described in the Methods. Lung adenocarcinoma, *n* = 24; normal adjacent lung tissue, *n* = 8. ***P < 0.001. **e**–**j** Lung adenocarcinoma was induced by administration of Ad-Cre into the lung of Kras ^*LSL−G12D****/+***^ mice. Sixteen weeks later, lung tissues from the Kras ^*LSL−G12D****/+***^ mice administrated with Ad-Cre (**e**, lower panel) or without Ad-Cre as a control (**e**, upper panel) were shown. Scale bar, 500 μm. **f** Sections from areas of the tissues shown in **e** (as indicated in the figure) were analyzed by immunostaining with anti-PYCR1 and kindlin-2 antibody (**f**) and atomic force microscopy (**g**–**j**). Scale bar in **f** = 20 μm. Stiffness mapping of tissues are shown in **g**; force (nN) vs. indentation depth (nm) graph highlighting the raw data and fitting by the Sneddon model to extract the tissues’ elastic moduli (**h**). Quantitative analysis using histograms of Young’s modulus values in log-normal scale with a Gaussian distribution fit (**i**) and Young’s moduli (**j**, *n* = 6 distinct locations that are macroscopically distant from each other) of the health lung tissue, lung adenocarcinoma (LAC), and normal tissue adjacent to the tumor (NAT) were analyzed as described in the Methods. Data in **c**, **d**, **j** are presented as mean ± SEM using one-way ANOVA with Tukey–Kramer post-hoc analysis (**j**) or two-tailed unpaired Student’s t-test (**c**, **d**), ***P < 0.001

We next investigated the functions of the kindlin-2–PYCR1 complex in vivo. To do this, we first analyzed the levels of kindlin-2 and PYCR1 in human lung adenocarcinoma by immunohistochemical staining. Both kindlin-2 and PYCR1 levels were markedly increased in cancerous tissues compared with those in normal tissues adjacent to lung adenocarcinoma (Fig. 8a–d). To confirm this, we analyzed the levels of kindlin-2 and PYCR1 in Kras^*G12D*^-induced lung adenocarcinoma in mice (Fig. 8e). Again, the levels of kindlin-2 and PYCR1 were markedly increased in Kras^*G12D*^-induced lung adenocarcinoma compared with those in normal mouse lung tissues (Fig. 8f). Atomic force microscopic and immunohistochemical analyses showed that regions of tumors with elevated levels of kindlin-2 and PYCR1 exhibited greater ECM tissue stiffness compared with those in normal regions adjacent to the tumors or healthy lung tissue (Fig. 8g–j). Collectively, these results suggest that the levels of kindlin-2 and PYCR1 are elevated, which are correlated with increased stiffness in lung adenocarcinoma in vivo.

### Kindlin-2 regulates fibrosis and tumor growth in vivo

**Fig. 9:**
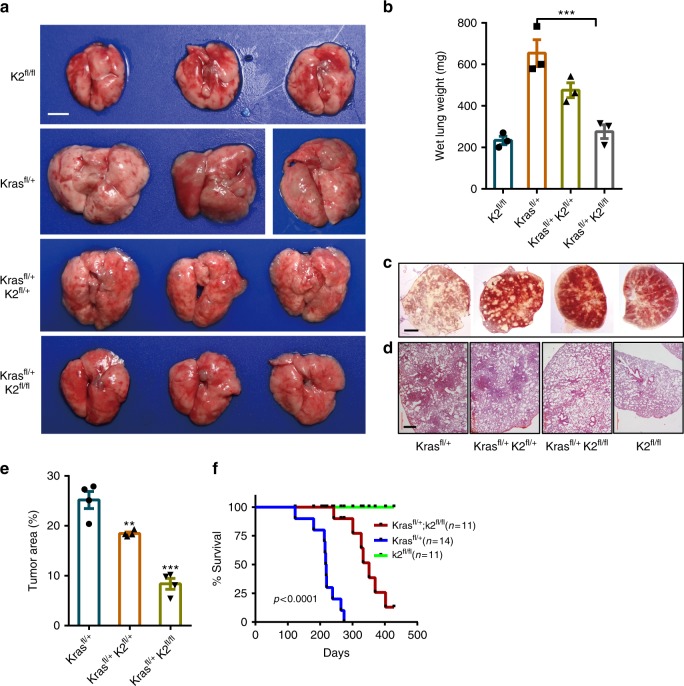
Ablation of kindlin-2 inhibits Kras^*G12D*^-induced lung tumorigenesis and reduces the mortality rate. **a**–**e** The lung of the mice (as indicated in the figure) was administrated with Ad-Cre and analyzed 16 weeks later. The gloss morphology of the lung was observed (**a**). Scale bar = 1 cm. The lung tissues from the mice were weighed (**b**, 3 mice for each group). The lung was pressed onto two slides and observed under microscopy for analyses of lung tumors (**c**). Scale bar = 0.5 cm. Sections of the lung tissues from the mice were analyzed by HE staining (**d**). Scale bar = 200 μm. Tumor area was quantified as described in the Methods (**e**, 4 mice for each group). **f** Kaplan–Meier survival curve of Kras^*LSL−G12D/+*^(Kras ^*fl/+*^) mice (*n* = 14) vs. Kras^LSL*−*G12D/+^; kindlin-2^*fl/fl*^ (Kras ^*fl/+*^; K2^*fl/fl*^) mice (*n* = 11) for up to 428 days post-administration of Ad-Cre. Data in **b** and **e** present mean ± SEM (*n* = 3) using one-way ANOVA with Tukey–Kramer post-hoc analysis, **P < 0.01; ***p < 0.001. Kaplan–Meir survival analysis in **f** was determined by log-rank test

**Fig. 10:**
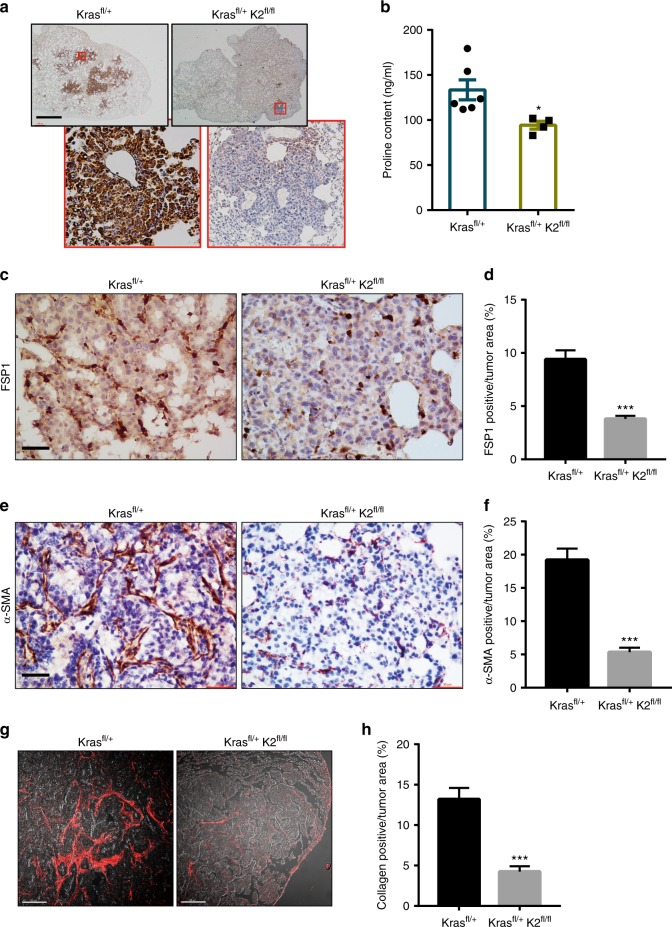
Ablation of kindlin-2 in lung adenocarcinoma substantially reduces PYCR1 and proline levels, and fibrosis in vivo. **a**–**h** The lung of the mice (as indicated in the figure) was administrated with Ad-Cre and analyzed 16 weeks later. Sections of the lung tissues from the mice (as specified in the figure) were analyzed by immunostaining with anti-PYCR1 antibody (**a**). Scale bar = 300 μm. The proline levels in the lung tissues were analyzed as described in the Methods (**b**, Kras ^*fl/+*^ group *n* = 6; Kras ^*fl/+*^; K2^*fl/fl*^ group *n* = 4). Sections of the lung tissues from the mice (as specified in the figure) were analyzed by immunostaining with an antibody for fibroblast-specific protein 1 (FSP1), a marker of fibroblasts (**c**). Scale bar = 20 μm. (**d**) The percentages of FSP1-positive areas among total tumor areas were quantified (at least 25 fields from 4 mice were counted). Sections of the lung tissues from the mice (as specified in the figure) were analyzed by immunostaining with an antibody for myofibroblast marker α-SMA (**e**). Scale bar = 20 μm. **f** The percentages of α-SMA-positive areas among total tumor areas were quantified (at least 35 fields from 4 mice were counted). Sections of the lung tissues from the mice (as indicated in the figure) in which collagen fibers were analyzed by second harmonic generation (SHG) with multiphoton microscopy are shown (**g**). Scale bar in **g**, 100 μm. The percentages of collagen matrix-positive areas among total tumor areas were quantified (**h**, at least 30 fields from 4 mice were counted). Data in **b**, **d**, **f**, **h** represent mean ± SEM. Statistical significance was calculated using two-tailed unpaired Student’s *t*-test, *P < 0.05; ***P < 0.001

The marked increase of the kindlin-2 and PYCR1 levels in lung adenocarcinoma, together with the molecular and cellular studies showing that kindlin-2 interacts with PYCR1 and promotes PYCR1 and proline levels and cell proliferation, beg the question as to whether kindlin-2 is involved in promoting lung tumorigenesis in vivo. To test this, we crossed kindlin-2^*fl/fl*^ mice with Kras^*LSL−G12D****/+***^(Kras^*fl/+*^) mice and obtained Kras^*LSL−G12D****/+***^; kindlin-2^*fl/fl*^ and Kras^*LSL−G12D****/+***^; kindlin-2^*fl/+*^ mice, respectively. Adenovirus encoding Cre (Ad-Cre) was administrated into the lung to induce the expression of Kras^*G12D*^ and inactivation of the kindlin-2 gene. Kindlin-2^*fl/fl*^ mice and Kras^*fl/+*^ mice were used in parallel experiments as negative and positive controls, respectively. As expected, expression of Kras^*G12D*^ markedly induced lung tumor formation in Kras^*LSL−G12D****/+***^ mice (Fig. 9a–e). However, the tumors formed in Kras^*LSL−G12D****/+***^; kindlin-2^*fl/+*^ mice administrated with Ad-Cre were significantly smaller compared with those in Kras^*LSL−G12D****/+***^ mice administrated with Ad-Cre (Fig. 9a–e). Furthermore, the inhibition of tumor formation was even more dramatic in Kras^*LSL−G12D/+*^; kindlin-2^*fl/fl*^ mice administrated with Ad-Cre (i.e., kindlin-2 conditional KO mice) (Fig. 9a–e). Consistent with the studies in lung cancer cells in culture, the levels of PYCR1 (Fig. 10a) and proline (Fig. 10b) were significantly reduced in response to conditional KO of kindlin-2, confirming that kindlin-2 is critical for control of PYCR1 and proline levels in vivo. Interesting, although abundant fibroblasts (Fig. 10c–f) and collagen matrix (Fig. 10g, h) were detected in the lung tissues of the Kras^*LSL−G12D****/+***^ mice administrated with Ad-Cre, much lower levels of fibroblasts (Fig. 10c–f) and collagen matrix (Fig. 10g, h) were detected in the lung tissues of the Kras^*LSL−G12D****/+***^; kindlin-2^*fl/fl*^ mice administrated with Ad-Cre. Finally, we determined the effect of conditional KO of kindlin-2 on the mortality rate of the mice in response to Kras^*G12D*^ activation. Kras^*LSL−G12D****/+***^ mice administrated with Ad-Cre had a median survival time of 218 days and all the mice died by day 274 after Kras^*G12D*^ activation. KO of kindlin-2 significantly reduced the mortality rate of the mice with Kras^*G12D*^ activation-induced lung adenocarcinoma. Specifically, the Kras^*LSL−G12D****/+***^; kindlin-2^*fl/fl*^ mice administrated with Ad-Cre had a median survival time of 333 days, with 4 out of 11 of the mice remained alive by day 428 (Fig. 9f).

## Discussion

PYCR1, which is localized primarily in mitochondria and catalyzes the NAD(P)H-dependent reduction of Δ1-pyrroline-5-carboxylate to proline, has a central role in proline metabolism and signaling^1,3^. Our studies described here identify kindlin-2, a component of the cell–ECM signaling pathway, as a key binding partner of PYCR1. Using both biochemical and imaging approaches, we show that kindlin-2 is present not only in focal adhesions but also in the mitochondria, where it forms a complex with PYCR1 and regulates the level and function of PYCR1. Specifically, depletion of kindlin-2 markedly reduces the PYCR1 level and concomitantly inhibits proline synthesis, increases ROS production and apoptosis, and inhibits cell proliferation. The loss-of-kindlin-2-induced inhibition of proline synthesis and cell proliferation can be reversed to a large extent by re-expression of PYCR1, illustrating the importance of PYCR1 in kindlin-2-mediated regulation of proline synthesis and cell proliferation.

In addition to identifying kindlin-2 as a key binding partner and regulator of PYCR1, our studies show that kindlin-2 mitochondrion localization, its complex formation with PYCR1, and consequently proline synthesis are regulated by cell mechano-environment. Specifically, kindlin-2 mitochondrion localization and its complex formation with PYCR1 are enhanced in cells adhered to stiff ECM and diminished in those adhered to soft ECM. Consistent with this, the levels of PYCR1 and proline and cell proliferation are increased in response to ECM stiffening. Of note, the ECM stiffening-induced increases of the PYCR1 and proline levels and cell proliferation are abolished in the absence of kindlin-2. Thus, the kindlin-2–PYCR1 interaction can sense the alteration of cell mechano-environment (i.e., ECM stiffening) and links it to proline synthesis and cell proliferation. Given the prominent roles of proline metabolism in regulation of energy production, protein synthesis, redox balance, and intracellular signaling^1–5^, the mechano-responsive kindlin-2–PYCR1 complex likely serves as a key axis in mechano-regulation of proline metabolism, signaling, and cell proliferation.

Metabolic reprogramming, which allows cancer cells to survive, proliferate and disseminate under altered microenvironment, is a hall marker of cancer^2,43^. Using multiple approaches including cultured lung adenocarcinoma cells, tissues from human patients with lung adenocarcinoma and a mouse model of lung tumorigenesis, we provide strong evidence suggesting that the kindlin-2-PYCR1 axis plays a crucial role in lung tumorigenesis. While metabolism is clearly influenced by cell microenvironment, alterations of metabolism can also have profound impacts on cell microenvironment including ECM. In this regard, it is worth noting that nearly 25% of amino acids incorporated into collagen, a major component of ECM, are proline. Thus, alteration of proline metabolism could potentially influence collagen synthesis and ECM remodeling. Consistent with elevation levels of PYCR1 in many types of human tumors^3,5–7,10–13^, increased collagen matrix formation and ECM stiffening are characteristics of human tumors^17,22–28^, which, through both biochemical and mechanotransduction pathways, contribute to the survival, proliferation, and dissemination of cancer cells. In the current study, we show that genetic ablation of kindlin-2 is sufficient to inhibit the increases of the levels of PYCR1, proline, and collagen matrix in lung adenocarcinoma and reduces tumor burden and mortality rate in vivo (Fig. 9, 10). Thus, the increased expression of kindlin-2 detected in lung adenocarcinoma is functionally significant, in that it not only functions in intracellular signaling but also promotes collagen matrix formation and consequently ECM stiffening, which in turn promotes the formation of the kindlin-2–PYCR1 complex, proline synthesis, and cell proliferation. Thus, there appears to be a positive cycle between the kindlin-2–PYCR1 complex formation and collagen synthesis/ECM stiffening that promotes lung tumorigenesis. Although immunohistochemical analyses show that kindlin-2 level is increased in human lung adenocarcinoma (Fig. 8a, c) and KO studies in mice demonstrate that increase of kindlin-2 level promotes lung adenocarcinoma progression and increases mortality rate (Fig. 9), how kindlin-2 level is increased remains to be determined. We have analyzed kindlin-2 (FERMT2) mRNA level using GEPIA (Gene Expression Profiling Interactive Analysis, http://gepia.cancer-pku.cn)^44^ and the results showed no statistically significant relationship between kindlin-2 mRNA level and the overall survival rate, albeit patients with higher mRNA levels of kindlin-2 tended to have worse overall survival rates (Supplementary Fig. 5). Thus, it is likely to be that a posttranscriptional mechanism is largely responsible for the increase of kindlin-2 protein level in lung adenocarcinoma. In this regard, it is worth noting that the level of PYCR1 is also significantly increased in human lung adenocarcinoma (Fig. 8b, d); analyses of PYCR1 mRNA level in human patients with lung adenocarcinoma also failed to reveal a significant correlation between increase of PYCR1 mRNA level and that of mortality rate, albeit patients with higher mRNA levels of PYCR1 tended to have worse overall survival rates (Supplementary Fig. 5). This is consistent with our finding that kindlin-2 promotes PYCR1 level at the protein rather than mRNA level (Fig. 4).

Finally, although our studies provide strong evidence for a crucial role of the kindlin-2–PYCR1 signaling axis in liking mechano-environment to proline metabolism and signaling in cancer cells and tumor growth in vivo, our studies do not rule out the possibility that kindlin-2 may also contribute to the progression of lung adenocarcinoma through other mechanisms. In this regard, it is worth noting that KO of kindlin-2 significantly reduces the number of stromal fibroblasts in lung adenocarcinoma (Fig. 10c–f), which likely contributes to the downregulation of collagen matrix deposition that is associated with the loss of kindlin-2. Although the current study focuses on the kindlin-2–PYCR1 signaling axis in lung adenocarcinoma cells, this signaling axis may also function in other cell types. Consistent with this, kindlin-2 also interacts with PYCR1 in mesenchymal stem cells (MSCs), and similar to what we found in lung adenocarcinoma cells, the kindlin-2–PYCR1 interaction in MSCs is enhanced in response to ECM stiffening (Supplementary Fig. 6). Thus, it is likely to be that kindlin-2, through its interactions with ECM adhesion receptors such as integrins^45,46^, actin cytoskeletal regulatory proteins such as ILK^30,47^, paxillin^48^, Arp2/3^49^, and MLCK^37^, and metabolic enzymes such as PYCR1 (the current study), promotes ECM adhesion, actin cytoskeletal contraction, cell survival, proliferation, metabolic reprograming, and collagen matrix deposition in both lung adenocarcinoma cells and associated fibroblasts, which collectively contribute to the progression of lung adenocarcinoma. Consistent with crucial roles of kindlin-2 in driving tumor fibrosis and growth, ablation of kindlin-2 effectively inhibits lung fibrosis and tumor growth in vivo (Fig. 9, 10). Thus, therapeutic inhibition of the kindlin-2 signaling pathways may provide an attractive approach for intervening lung cancer progression.

## Methods

### Mice

Kindlin-2^*fl/fl*^ transgenic mice were generated as described^36^. Kras^*LSL−G12D****/+***^ mice were bought from the Jackson Laboratory. All mouse work was performed with the approval of the Institutional Animal Care and Use Committee, Southern University of Science and Technology.

### Mouse genotyping and recombinant allele detection

Genotyping of LSL-Kras^*G12D*^ and floxed kindlin-2 alleles was performed by PCR using oligonucleotide primers as described previously^36,50^. The recombinant alleles were analyzed using genomic DNA extracted from the tips of mouse tails.

### Ad-Cre infection of mouse lung

To activate Kras^*G12D*^ in the lung, intranasal administration of Ad-Cre was performed as described^51^. Briefly, age-matched mice (6–8 weeks old) of both sexes (as specified in each experiment) were anesthetized by intraperitoneal injection of 20 mg ml^*−*1^ avertin at room temperature. Ad-Cre:CaPi coprecipitates were prepared by mixing Ad-Cre (purchased from BAC Biological Technology Co., Ltd) at the dose of 3 × 10^7^ pfu in a total volume of 125 μl and CaCl_2_ (at a final concentration of 10 mM CaCl_2_). The Ad-Cre:CaPi coprecipitates were loaded in a pipet tip and administered nasally using two 62.5 μl instillations with a 5 min interval.

### Immunohistochemical staining

Mouse lung organs (as specified in each experiment) at 16 weeks after Ad-Cre infection were isolated after 10% formalin perfusion, fixed in 10% formalin, and embedded in paraffin as described^37^. Sections that were 5 μm thick were cut for hematoxylin and eosin (H&E) staining, and examined under a microscope. Immunohistochemistry was performed using the MaxVision^TM^ HRP-Polymer anti-Mouse/Rabbit IHC Kit (MXB Biotechnologies) with antibodies against kindlin-2 (clone 3A3.5^37^), α-Smooth Muscle (α-SMA, Sigma, A2547), FSP1 (Proteintech,16105-1-AP), or PYCR1 (Proteintech, 13108-1-AP). Sections were developed with 3,3′-Diaminobenzidine and counterstained with hematoxylin. FSP1^+^, α-SMA^+^ cells were measured using Image-Pro Plus software version 6 (Media Cybernetics, Silver Spring, MD). Sections of four lung tissues from four individual mice in each experimental group were taken for analysis. The sizes of the study tumor areas and the integrated optical density (IOD) of FSP1^+^, α-SMA^+^ were manually counted in three randomly selected fields per slide under a × 40 objective.

Lung adenocarcinoma tissue microarray (LC953) was purchased from Alenabio (Xian, China). A detailed description of the clinical information (age, sex, grade, stage, etc.) of the human specimens was provided by Alenabio (Xian, China) and is included in Supplementary Table S1. Molecular data (e.g., KRAS mutational status) for these tissue microarray samples are unavailable. Kindlin-2 and PYCR1 immunostaining were quantified using Image-Pro Plus software version 6 (Media Cybernetics, Silver Spring, MD) as described^52^. Briefly, digital images (TIFF format) close to the center of each tissue core were captured using a digital camera (DS-Fi1c; Nikon) and NIS-Elements F Ver4.30.01 image analysis software (Nikon) with a × 40 objective. Images of all tissue cores were acquired in the same session using a constant set of microscope and imaging software parameters. The images were subjected to optical density analysis using Image-Pro Plus software. Intensity range was selected based on the image histogram, with intensity and saturation set at maximum, and hue set at a range where most of the brown diaminobenzidine tetrahydrochloride hydrate color was selected, whereas the blue-counterstained nuclei were excluded. These settings were saved and subsequently applied to all of the images analyzed. After defining the region of interest, the mean optical density of the selected area (IOD per unit area) was determined using the software. The IOD per unit area represents the immunoreactivity of kindlin-2 or PYCR1 within the tumor tissue.

### Quantification of tumor areas

H&E-stained slides were scanned at × 2.5 objective magnification with a digital camera (DS-Fi1c; Nikon) and NIS-Elements F Ver4.30.01 image analysis software (Nikon). Lung tumor areas were quantified using Image J software in manual measurement mode.

### Cell culture, viral vector generation, and infection

Human A549 (ATCC® CCL-185™) and NCI-H358 [H-358, H358] (ATCC^®^ CRL-5807™) lung adenocarcinoma cells were cultured in Dulbecco’s modified Eagle’s medium (DMEM) supplemented with 10% fetal bovine serum (FBS) (Gibco-Invitrogen), 50 U ml^*−*1^ penicillin and streptomycin at 37 °C in 5% CO2. Prior to shipping each cell line, the ATCC performed cell line authentication and mycoplasma testing.

For generation of lentiviral vectors encoding kindlin-2 and control shRNAs, the pLKO.1, psPAX2 and pMD2.G vectors were from Addgene (plasmid #10878). The pLKO.1 vectors expressing short hairpin RNA (shRNA) targeting human kindlin-2 or scrambled shRNA (sh-NC) sequence were generated using the following sequences: Sh-Kindlin-2: 5′-AACAGCGAGAATCTTGGAGGC-3′; Sh-PYCR1: 5′- GCCCACAAGATAATGGCTA-3′; Sh-NC: 5'-ACGCATGCATGCTTGCTTT-3'. Lentiviruses encoding the above shRNAs were generated by co-transfection of 293T cells with pLKO.1 encoding the shRNAs, psPAX2 and pMD2.G vectors. To generate DNA expression vectors (i.e., pLVX-K2, pLVX-mCherry-K2, pLVX-3FLAG-kindlin-2, pLVX-3FLAG-kindlin-2∆F1∆F3 and pLVX-3FLAG-PYCR1 vectors), complementary DNAs encoding the corresponding protein sequences were cloned into the pLVX-3FLAG or pLVX-mCherry vectors. Lentiviral expression vectors encoding kindlin-2, mCherry-kindlin-2, 3xFLAG-kindlin-2, pLVX-3FLAG-kindlin-2∆F1∆F3 or 3xFLAG-PYCR1 were co-transfected with psPAX2 and pMD2.G into 293T cells. After the cells were incubated at 37 °C, 5% CO2 for 24-48 h, the media contained lentiviral particles were harvested. For lentiviral or adenovirus infection, A549 cells were cultured in basal growth medium until 70% confluence and then replaced with fresh medium containing lentivirus or adenovirus (as specified in each experiment) at a multiplicity of infection (MOI) of 100 for 16 h. Lentiviral infections were carried out in the presence of 8 μg ml^*−*1^ polybrene.

### HMSCs isolation and culture

Human MSCs (hMSCs) were isolated from human placenta as previously described^53,54^. Briefly, term (38-40 weeks’ gestation) placentas from healthy donors were harvested with written informed consent and the procedure was approved by the Ethics Committee of Shenzhen Futian District Second People’s Hospital. The placental tissue was washed several times with cold phosphate-buffered saline (PBS) and then mechanically minced and enzymatically digested with 0.25% trypsin-EDTA for 30 min at 37 °C in a water bath. The digest was subsequently filtered, pelleted and re-suspended in a growth medium consisting of DMEM (Gibco-Invitrogen), 10% FBS (Gibco-Invitrogen) and antibiotics. Cells were seeded on cell culture dishes and medium was replaced every 2 days to reach 80% confluence.

### Generation of kindlin-2 KO A549 cells

Kindlin-2 KO A549 cells were generated with CRISPR/Cas9-mediated gene editing. Two guide RNA oligoes designed to target the sequence of 5′-ccctcagcacaaactgctccgcc-3′ and 5′-tcccaacatgaagtatgtgaagg-3′ located at the exon 1 of kindlin-2, respectively, were cloned into pSpCas9n (BB)-2A-GFP (PX461 containing cas9n was obtained from Dr Feng Zhang, Addgene plasmid #48140) via BbsI sites and transfected into A549 cells. Single GFP (green fluorescent protein)-positive cells were sorted into each wells of 96-well plate by FACS sorter (BD FACS AriaTMIII) for further propagation. Individual kindlin-2 KO colonies were examined and confirmed by DNA sequencing and western blotting. The kindlin-2 KO A549 cells were cultured as described above. For GM132 and leupeptin inhibition studies, Kindlin-2 KO A549 cells were treated with MG-132 (Selleck,10 μM) or leupeptin (Selleck,10 μM) for 12 h, and then collected and analyzed as specified in each experiment.

### Quantitative reverse transcriptase-PCR analysis

Total RNA was extracted from cells with TRIzol (Invitrogen) following the manufacturer’s instructions. First-strand cDNA was prepared by reverse transcription with Superscript II reverse transcriptase (Invitrogen) and oligo(dT) primers, and stored at 20 °C. Reverse transcriptase-PCR was performed using SYBR ® Premix Ex Taq™ II with an ABI 7500 QPCR System. As an internal control, levels of glyceraldehyde-3-phosphate dehydrogenase (GAPDH) mRNA were quantified in parallel with mRNAs of the target genes. Normalization and fold changes were calculated using the ∆∆Ct method. Primer sets are listed in Supplementary Table 2.

### Isolation of mitochondrial and cytosolic fractions

Mitochondrial and mitochondrion-free cytosolic fractions were prepared as we described^55^. Specifically, A549 cells were suspended in 5 mm Tris buffer (pH 7.4) containing 5 mm KCl, 1.5 mm MgCl_2_, 0.1 mm EGTA, 1 mm dithiothreitol, 1 mm phenylmethylsulfonyl fluoride, 5 μg ml^*−*1^ leupeptin, and 2 μg ml^*−*1^ aprotinin. The cell suspension was passed through a 22-gauge needle 20 times and centrifuged at 700 × *g* for 10 min at 4 °C to remove the nuclei. The supernatants were centrifuged at 12,000 × *g* for 15 min at 4 °C. The pellets (mitochondrial fraction) and supernatants (cytosolic fraction) were collected. Equal amount (10 μg) of the mitochondrial, cytosolic, and total cell lysates were analyzed by western blotting.

### Western blotting

Western blotting was performed as previously described^36,37^. For preparation of total cell lysates, cells were lysed in 1% SDS lysis buffer (25 mM Tris-HCl (pH 6.8), 50 mM dithiothreitol (DTT), 8% glycerin, 2.5% sucrose and 1% sodium dodecyl sulfate (SDS)). Equal amounts (10–40 μg per lane) of cell proteins were separated on 10% polyacrylamide gel and transferred onto a nitrocellulose membrane. Membranes were blocked for 1 h at room temperature in Tris-buffered saline (50 mM Tris-HCl, 150 mM NaCl, pH 7.4) containing 0.1% Tween 20 and 5% non-fat powdered milk, followed by overnight incubation at 4 °C with mouse anti-GAPDH (Abmart, M20006,1:5000), rabbit anti-PYCR1 (Proteintech, 13108-1-AP, 1:1000), rabbit anti-PYCR2 (Proteintech, 17146-1-AP, 1:1000), rabbit anti-PYCRL (absin, abs105382, 1:1000), rabbit anti-P5CS (Proteintech, 17719-1-AP, 1:1000), rabbit anti-PRODH (Proteintech, 22980-1-AP, 1:1000), mouse anti-kindlin-2 (clone 3A3.5^37^, 1:1000), rabbit anti-FAK (Santa Cruz, sc-557, 1:1000), mouse anti-phospho-FAK (Tyr397) (Millipore, 05-1140, 1:1000), mouse anti-tubulin (Development Studies Hybridoma Bank, Clone E7, 1:1000), or rabbit anti-PHB2 (Upsate Biotechnology, 07-234, 1:1000) antibodies. After washing and incubation with appropriate horseradish peroxidase-conjugated secondary anti-rabbit or mouse IgG antibodies (Jackson ImmunoResearch, #711-005-152 or #715-005-151, 1:10,000), blots were developed using an enhanced chemiluminescence kit (ECL Kit, Bio-Rad) and then exposed to X-ray film (Fuji film, #super RX-N-C). The images were scanned using an imaging scanning system (EPSON Scan; L365). Quantification of band intensities was performed with Image J. All western blotting images were cropped to optimize clarity and presentation. Uncropped scans of the western blotting images presented in the main figures of this manuscript are shown in Supplementary Fig. 7–9.

### Immunofluorescence

Cells (as specified in each experiment) were seeded on fibronectin-coated coverslips in 24-well plates (2 × 10^4^ cells/per well) and cultured overnight. Cells were fixed with 4% paraformaldehyde (PFA), washed three times with PBS, immersed in 0.1% Triton X-100 in PBS for 10 min at room temperature, and then washed three times with PBS again and incubated with mouse anti-kindlin-2 (clone 3A3.5,1:5000-10,000^37^), rabbit anti-PYCR1 (Proteintech, 13108-1-AP, 1:500), rabbit anti-Ki67 (Abcam, ab16667, 1:100), or rabbit anti-cleaved caspase-3 (CST, #9664, 1:50) antibodies at 4 °C overnight. Cells were then washed three times with PBS and incubated with the corresponding secondary antibodies (Alexa Flours, Invitrogen) at 1:500 dilution at room temperature. In some experiments, cells were co-cultured with MitoTracker™ Deep Red FM (Invitrogen, M22426) for 1 h before fixing. When indicated, cells were co-stained with DAPI (4,6-diamidino-2-phenylindole) for 30 min at room temperature. Images were acquired at 21 °C using an SP8 confocal fluorescence microscope (× 20 dry objective 0.7 numerical aperture (NA), × 40 dry objective 0.85 NA, or × 63 oil objective 1.4 NA; Leica) with Leica X Version:1.1.0.12420 image software. The number of Ki67-positive cells or cleaved caspase-3-positive cells was counted from five microscopic fields (from the center and four sides of the well at × 20 magnification) and quantified using Image J. At least 50 cells were counted in each experiment. The means ± SEM were calculated using one-way analysis of variance (ANOVA).

### Integrin internalization assay

Integrin internalization assay was performed based on the method described by Du et al.^42^ with minor modifications. Briefly, A549 cells grown on soft substrate were treated with or without 10 mM MBCD for 1 h. The cells were then incubated with an antibody for β1 integrin (1:300; Millipore, #MAB1987) at 4 °C for 45 min After washing three times, cells were incubated at 37 °C for 30 min, to allow integrin internalization. At the end of the incubation, cell surface antibodies were removed with acidic medium (pH 4.0). Internalized β1 integrin bound to the antibody was detected using fluorescein isothiocyanate-conjugated anti-mouse IgG antibodies (1:400; Abcam).

### Immunoprecipitation

Cells (as specified in each experiment) were collectedand homogenized in IP lysis buffer (P0013, Beyotime, China) supplemented with 1 mM phenylmethylsulfonyl fluoride (Sigma-Aldrich, Cat# 329-98-6) for 30 min at 4 °C, and pre-cleared with Protein A/G PLUS-Agarose (Santa Cruz, Cat# sc-2003) for 30 min. IP was performed overnight at 4 °C by incubation of the cell lysates containing equal amount of proteins (0.5–2 mg) with mouse anti-kindlin-2 antibody (clone 3A3.5^37^), rabbit anti-PYCR1 (Proteintech, 13108-1-AP), mouse anti-FLAG® M2 antibody (Sigma, F1804), or irrelevant mouse IgG (Santa Cruz, Cat# sc-2025) (as a negative control) as specified in each experiment. Antibodies and associated proteins were immunoprecipitated by incubation of the samples with Protein A/G PLUS-Agarose for 2 h, followed by washing once with the lysis buffer and twice with PBS. The samples were then subjected to western blotting using rabbit anti-PYCR1 (Proteintech, 13108-1-AP, 1:1000), rabbit anti-PYCR2 (Proteintech, 17146-1-AP, 1:1000), rabbit anti-PYCRL (absin, abs105382, 1:1000), rabbit anti-P5CS (Proteintech, 17719-1-AP, 1:1000), rabbit anti-PRODH (Proteintech, 22980-1-AP, 1:1000), mouse anti-phospho-serine (Millipore, clone4A4, 1:1000), rabbit anti-phospho-tryosine (CST, 8954S, 1:1000), rabbit anti-ubiquitin (Proteintech, 10201-2-AP, 1:1000), or rabbit anti-kindlin-2 (Proteintech, 11453-1-AP, 1:1000) antibodies.

### Nano LC-MS/MS analysis

Nano LC-MS/MS was carried out as we described previously^37^. Briefly, A549 cell lysates were immunoprecipitated with mouse anti-kindlin-2 antibody (clone 3A3.5^37^) or control mouse IgG (Santa Cruz, Cat# sc-2025). Each IP sample was then resolved in SDS loading buffer and denatured at 95 °C for 5 min. After protein separation by 10% SDS-polyacrylamide gel electrophoresis (PAGE) and staining with Brilliant Blue G-250, each lane of the gel was excised, cut into six slices, and incubated with 500 μl 50 mM ammonium bicarbonate (ABC) and 50% (vol/vol) acetonitrile (ACN) for destaining. The gel slices were dehydrated and rehydrated with 200 μl 100% ACN. Disulfide bonds were reduced with 200 μl of 10 mM DTT. The proteins were alkylated with 100 mM iodoacetamide and 50 mM ABC for 30 min at room temperature in the dark. The gel pieces were dried in a speed vacuum and incubated overnight at 37 °C with sequencing-grade modified trypsin (Promega, Fitchburg, WI) at an enzyme-to-protein ratio of 1:100 (wt/wt). The resulting peptides were extracted sequentially from the gel slices with 200 μl of 25 mM ABC and 200 μl 5% (vol/vol) formic acid (FA), 50% ACN by sonication for 20 min at each stage. After all supernatants were combined, the peptides were dried in a speed vacuum. The dried peptide mixtures were dissolved in 100 μl 1% FA and desalted using homemade C18-StageTips, which were prepared as described previously^56^. Finally, the eluted peptide samples were lyophilized to dryness and redissolved in 10 μl of 0.1% (vol/vol) FA in water for nano LC-MS/MS analysis. Peptide mixtures were analyzed by an Orbitrap Fusion mass spectrometer (Thermo Fisher Scientific) coupled with an Easy-nLC 1000 (Thermo Fisher Scientific) ultrahigh-pressure LC pump. The LC separation system consisted of a trap column (100 μm i.d. × 4 cm) and an analytic column with integrated spray tip (75 μm i.d. × 20 cm), both packed with 3 μm/120 Å ReproSil-Pur C_18_ resins (Dr Maisch GmbH, Ammerbuch, Germany). The buffers used for separation were 0.1% FA in water and 0.1% FA in ACN. Half of the obtained samples were first loaded onto the trap column at a flow rate of 2 μl/min and then separated by the analytic column at a flow rate of 300 nl/min. The gradient was set as follows: from 3% to 7% ACN in 2 min, from 7% to 22% ACN in 50 min, from 22% to 35% ACN in 10 min, from 35% to 90% ACN in 2 min, holding at 90% ACN for 6 min, declining to 3% ACN in 2 min, and holding at 3% ACN for 13 min. Full MS scans were performed in an Orbitrap mass analyzer over m/z range of 350–1550 with a mass resolution of 120,000. The MS data have been deposited in MassIVE with accession code MSV000083291 [ftp://massive.ucsd.edu/MSV000083291].

### GST-fusion protein pulldown assay

For generation of GST-fusion proteins containing wild type or mutant forms of kindlin-2, the corresponding cDNA sequences were cloned into pGEX-4T-1 vector. GST and GST-fusion proteins were expressed in *Escherichia coli* strain BL21 (DE3) cells and purified with Glutathione-Sepharose 4B matrix (GE Healthcare) following the manufacturer’s protocol. PYCR1 was expressed with His tag at the N-terminal in *E. coli* and purified with nickel resin (Sangon). Purified proteins were resolved by SDS-PAGE to verify their size and purity. In pulldown assays, GST or GST-fusion proteins bound to glutathione-Sepharose beads were incubated with purified His-tagged PYCR1 for 2 h at 4 °C. The beads were washed three times with buffer (20 mM Tris pH 7.5, 150 mM NaCl and 0.1% Triton) and analyzed by SDS-PAGE. His- and GST-tagged proteins were detected by western blotting with mouse anti-His (Tiangen, AB102, 1:1000) and anti-GST (Transgen, HT601, 1:1000) antibodies, respectively.

### PYCR1 enzyme activity

The dehydrogenase activity of PYCR1 was assayed as previously described^57^. Briefly, the enzyme reaction was initialized by adding PYCR1 (1.5 μl, 2 mg ml^*−*1^) with or without GST-Kindlin-2 (6 μl, 0.5 mg ml^*−*1^) to 200 μl of reaction buffer containing 300 mM Tris-HCl (pH 9.0), 1 mM NAD+ (Sangon), and 0.22 mM 3,4-dehydro-l-proline (Sigma). Using the mM extinction coefficient of NADH (6.22), initial rates of product formation were calculated as the increase of absorbance at 340 nm min^*−*1^ from the first 10 s of a 5 min recording period (EnSpire™ Multimode Plate Reader, PerkinElmer, USA). All analyses were repeated at least three times at room temperature. A sample that included both substrates without PYCR1 served as a negative control.

### Analysis of ROS

DHE (Dihydroethidium, Beyotime, China) fluorescence probe was used to quantify the level of ROS according to the manufacturer’s instruction. Briefly, A549 cells were washed three times with PBS and then loaded with DHE probe (10 μM) and incubated at 37 °C for 30 min. The cells were washed three times with PBS and fixed in 4% PFA at room temperature for 30 min. At the end of incubation, the cells were washed three times with PBS and stained with Hoechst at room temperature for 15 min. The cells were observed under a fluorescent microscope (Olympus IX73, Olympus Co., Ltd). The fluorescence intensity of five microscopic fields (from the center and four sides of the well at × 20 magnification) of each group was measured and analyzed with Image-pro plus software. The relative fluorescence intensity was taken as the average of values from three repeated experiments.

### Collagen-I-coated hydrogels with different stiffness

A549 cells were plated on collagen-I-coated polyacrylamide hydrogels following a previously described protocol^37,40,41^. Briefly, glass coverslips were activated successively with 100 mM NaOH for 5 min, 3-aminopropyltri-methoxysilane (Sigma-Aldrich, Cat# 281778) for 5 min, and 0.5% glutaraldehyde (Sigma-Aldrich, Cat# G7776) for 30 min Hydrogels with different stiffness were prepared with two pre-polymer solutions containing different acrylamide/bis-acrylamide ratios. The polyacrylamide sheets were assembled on the activated coverslips upon adding of 0.5% ammonium persulfate (Sigma-Aldrich, Cat# 09830) and 0.005% TEMED, and subsequently activated by incubation with 1 mM Sulfo-SANPAH (Thermo Scientific, Cat# 22589) under 452 nm UV light for 5 min. The activated polyacrylamide sheets were then coated with 0.3 mg ml^*−*1^ Collagen-I at 4 °C overnight before seeding with A549 cells at a density of 1.5 × 10^5^ cells on each 7.5 cm coverslip.

### Measurement of proline level

Cells (as specified in each experiment) were cultured with basic DMEM medium in the absence of FBS for 24 h. Cells (4 × 10^6^) were lysed with PBST (1% Triton X-100 in PBS) and the cellular debris were removed by centrifugation (9000 × *g*). The supernatants were transferred to a boiling water bath and intracellular amino acids were extracted by boiling for 10 min. After centrifugation (5 min, 4 °C, 15,000 × *g*), the proline level in the supernatant was determined as described^58^. Briefly, 200 μl of the supernatant was incubated with 400 μl of 1.25 % ninhydrin (0.125 g of ninhydrin dissolved in 6 ml of glacial acetic acid and 4 ml distilled water) for 20 min at 100 °C, followed by recording absorbance of the proline-ninhydrin condensation product in the reaction mixture itself at 508 nm. A standard curve ranging in concentrations of 0–500 ng ml^*−*1^ proline was generated and used for determination of proline concentrations of the samples. For measurement of proline levels in the lung tissues, the same lobes of the lung tissues from the mice (as specified in each experiment) were collected. The level of proline in the lung tissues was quantified as above.

### Fluorescence lifetime imaging microscopy

Fluorescence lifetime imaging microscopy (FLIM)-FRET was performed to examine the interaction of kindlin-2 with PYCR1 in cells. To do this, cells were transfected with plasmids of FRET sensor pairs mClover2-N1 and mRuby2-C1 (Plasmid #54538 and #54768 respectively, Addgene). Twenty-four hours after transfection, the cells were fixed with 4% PFA and mounted on coverslips with mounting medium (Invitrogen, P36934) for FLIM imaging. FLIM-FRET was analyzed with a confocal time-correlated single photon counting (TCSPC) system, in which a compact FLIM upgrade kit (PicoQuant, Germany) was integrated into a Nikon A1R laser scanning confocal system (Nikon, Japan). While acquiring, picosecond-pulsed diode laser beam (LDH-P-C-485B, PicoQuant, Germany) was guided toward the objective and then excited the FRET sensors as specified in individual experiments, the emitted photons was guided through a 0.9 Airy unit pinhole as instructed by the system developer (PicoQuant, Germany) and was detected with a single-photon-sensitive detector (PMA hybrid, PicoQuant, Germany) with a bandpass filter of 520/20 nm (Semrock, USA). Time-resolved recordings of emitted photons were performed in the TCSPC mode using a TimeHarp 260 PICO board (PicoQuant, Berlin, Germany). In all experiments, the laser power was adjusted to achieve average photon counting rates significantly below the maximum counting rates allowed by TCSPC electronics, to avoid pile-up effects. Images were acquired by xy-Galvano scanning built in the Nikon A1R system (Nikon, Japan). All photons collected in regions of interest associated with kindlin-2 were used to form a global histogram for luminescence decay fitting to evaluate the fluorescence lifetime of the donor of FRET sensor and the FRET efficiency. Data acquisition and analysis were performed by the SymPhoTime64 software version 1.5 (PicoQuant, Berlin, Germany). Average lifetimes were calculated from the mean of all pixels measured within each image/cell and pooled from multiple experiments for statistical analysis. All histogram data are plotted as mean FRET efficiency from > 15 cells per sample. Each experiment was repeated at least three times.

### Multihoton microscopy

Mouse lung organs (as specified in each experiment) at 16 weeks after Ad-Cre infection were isolated after 10% formalin perfusion, fixed in 10% formalin, and embedded in paraffin as described^37^. Sections that were 5 μm thick were de-paraffinized and rehydrated. After de-paraffinization and re-hydration, thin lung sections were mounted on coverslips with Neutral Balsam (Yeasen, 36313ES60) for Multihoton microscopy (MPM), as MPM is able to specifically image collagen-I fibers by second harmonic generation (SHG)^59–62^. MPM was performed with a FVMPE-RS MPM system (Olympus, Japan) based on a Olympus IX83 inverted microscope, which was equipped with a femtosecond-pulsed Ti:Sa laser (Mai Tai DeepSee, Spectra-physics, USA). Emitted signals were collected using an apochromat objective (× 20/NA 0.75/WD 0.6, Olympus, Japan) and detected with two non-descanned photomultipliers and one camera (transmission detector). When imaging, the excitation laser was tuned to 960 nm and collagen in lung sections was visualized by the SHG of the excitation laser in backscattering mode. Emission signals were separated by a dichroic mirror and two band-pass filters (505DCXR, 480/40, and 540/40, respectively, Chroma Technology, USA). Collagen signals by SHG was collected in the 480/40 channel, whereas the 540/40 channel was to record autofluorescence (AF) as a result of sample preparation. Merged SHG/AF images were generated to identify collagen signals. Images of *z*-stacks were acquired and their maximum-intensity projections were generated to quantify the ration of collagen amount to lung tumor size using an image analysis software (Imaris, Bitplane, USA) as above α-SMA^+^ analysis.

### Nanomechanical measurement of tissues

The local elasticity of lungs tissues (healthy, cancer, and normal adjacent lung tissue) from mice were probed with a commercial AFM Dimension Icon (Bruker) in force volume (FV) mechanical imaging mode. In a FV experiment, a force curve (FC) is obtained by vertically indenting the AFM-tip at every point of a pre-designed regular matrix in the selected area, which yields the local mechanical response at each location. The morphology is reconstructed from the ensemble of the recorded FCs, thereby producing a 1:1 correspondence between the morphology and the map of the mechanical properties. A series of six to seven FV measurements were performed in different macroscopic positions, to improve the statistical reliability of the experiments. All samples were imaged while they were immersed in PBS solution at room temperature (*T* = 23 °C). We used a standard sharp indenter model DNP (Bruker) with nominal averaged semi-angle aperture *θ* = 19°. The elastic spring constant, *k* = 0.57 N/m, was calibrated in air and water using the standard thermal noise method integrated in the machine^63^. Subsequently, the performance was improved using Standardized Nanomechanical Atomic Force Microscopy Procedure with benchmarked polydimethylsiloxane samples^64^. Young’s moduli were evaluated by data analysis performed with a custom software “AFMech Suite” environment with a routine fully described in Galluzzi et al.^65–68^. Briefly, for each FV measurement, single FCs were pre-processed to obtain FCs (*F* [nN]) vs. indentation (*δ* [nm]) for each indentation. After the pre-processing, the indentation curves were fitted using the Sneddon model (with the Bilodeau approximation for four-sided pyramids instead of a conical indenter) for sharp probes^69,70^:

$$F = 0.7453\frac{{E\tan \theta }}{{\left( {1 - \nu ^2} \right)}}\delta ^2\;\left( \mathrm{Bilodeau} \right)$$

where *E*, *ν*, and *θ*, represent the local Young’s modulus, the Poisson ratio (0.5 for uncompressible hydrogels), and the averaged half-opening angle of the four-sided pyramidal indenter, respectively. We used the logarithmic Young’s moduli values to build the mechanical map and the collective histogram for statistical analysis. For a single FV measurement, the error associated must consider this calibration error and the variability of Young’s moduli in the FV area, i.e., the width of log normally distributed values. The final error characteristics depend on the error of a single FV and its variation due to measurements in different macroscopic locations on the same sample (six distinct locations macroscopically distant for each region).

### Statistical analysis

Data are presented as means ± SEM. Statistical analysis was performed using Student’s *t*- test (two-tailed) or one-way ANOVA with Tukey’s post-hoc test by GraphPad Prism (version 7). Survival functions were plotted using the Kaplan–Meier method and comparison of survival functions was performed by the log-rank test. A *p*-value < 0.05 was considered significant.

### Reporting summary

Further information on experimental design is available in the Nature Research Reporting Summary linked to this article.

## Supplementary information

Supplementary Information

Reporting Summary

## Data Availability

All relevant data are available from the authors. The mass spectrometry data have been deposited in MassIVE with accession code MSV000083291. [ftp://massive.ucsd.edu/MSV000083291]. Uncropped western blottings for Figs. 1a–i, 2d, 3a, 4a–l, 5a, 6a–m, and 7a–f are provided in Supplementary Figs. 7-9.
