# Supplementary material for: Kindlin-2 links mechano-environment to proline synthesis and tumor growth
Source: Nat Commun. 2019 Feb 19;10:845. doi: 10.1038/s41467-019-08772-3 (PMC6381112; doi:10.1038/s41467-019-08772-3)
Supplement: Supplementary file 1 — Supplementary Information [file 41467_2019_8772_MOESM1_ESM.pdf]

Kindlin-2 links mechano-environment to proline synthesis and tumor growth

Guo et al.

Supplementary information

Supplementary Figures

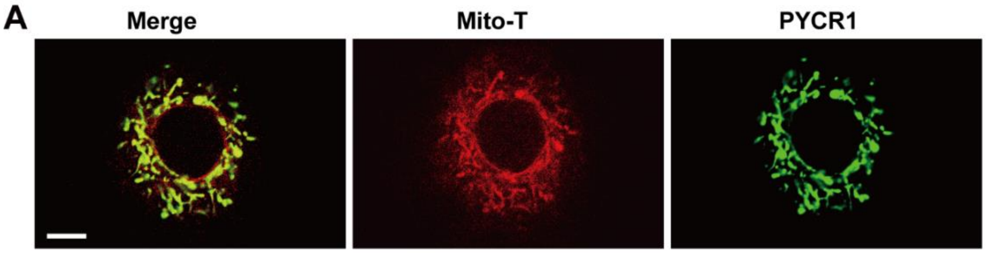

Supplementary Fig. 1

Supplementary Figure 1. PYCR1 is localized in mitochondria

A549 cells were labeled with MitoTracker™ Deep Red FM for 1 hour before fixing, and then stained with anti-PYCR1 antibody and observed under confocal microscopy. The MitoTracker (Mito-T, red) and anti-PYCR1 antibody (green) staining and merged image are shown. Bars,10 μm.

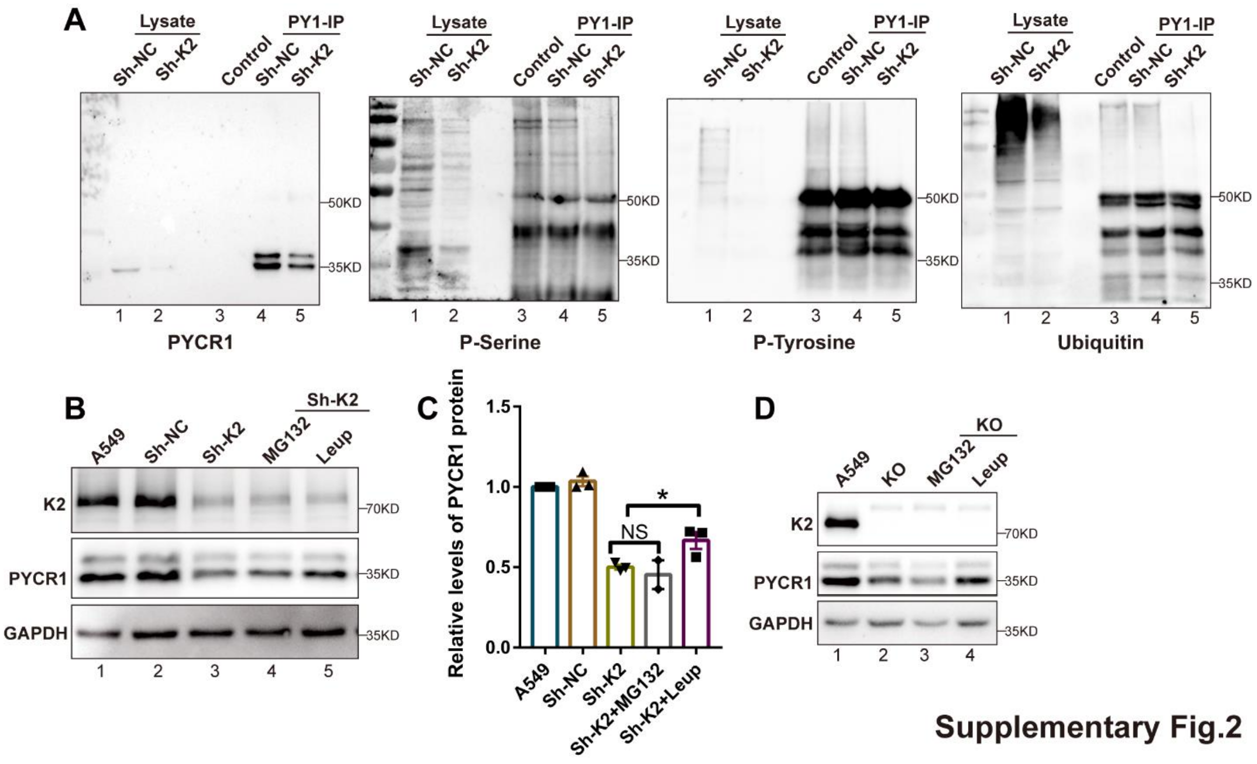

Supplementary Figure 2. Treatment of cells with leupeptin partially reverses the reduction of PYCR1 level induced by the depletion of kindlin-2

(A) A549 cells were infected with kindlin-2 shRNA (Sh-K2) lentivirus or control (Sh-NC) lentivirus for 4 days, lysed and analyzed by IP with monoclonal anti-PYCR1 antibody or irrelevant mouse IgG (as a control) as described in the Methods. The cell lysates (lane1 and 2), control IgG (lane 3) and anti-PYCR1 immunoprecipitates (PY1-IP, lane 4 and 5) were analyzed by Western blotting with PYCR1, phospho-serine, phospho-tyrosine or ubiquitin antibodies as indicated. (B) A549 cells were infected with kindlin-2 shRNA (Sh-K2) lentivirus or control (Sh-NC) lentivirus for 4 days and treated with MG132 (10 μM) or leupeptin (10 μM) for 12 hours. Cells were analyzed by Western blot with antibodies recognizing kindlin-2, PYCR1 or GAPDH (as a loading control). (C) Protein levels of PYCR1 relative to that of GAPDH were quantified by densitometric analyses of Western blots. The levels of PYCR1 in Sh-NC, Sh-K2,

Sh-K2+MG132 or Sh-K2+leupeptin cells were compared to that of A549 cells (normalized to 1). The bars of Sh-NC, Sh-K2, and Sh-K2+leupeptin represent means ± SEM from three independent experiments. The bar of Sh-K2+MG132 represents mean ± SEM from two independent experiments. *P<0.05. NS, no significance. (D) Kindlin-2 KO A549 cells were treated with MG132 (10 μM) or leupeptin (10 μM) for 12 hours and then analyzed by Western blot with antibodies recognizing kindlin-2, PYCR1 or GAPDH (as loading control).

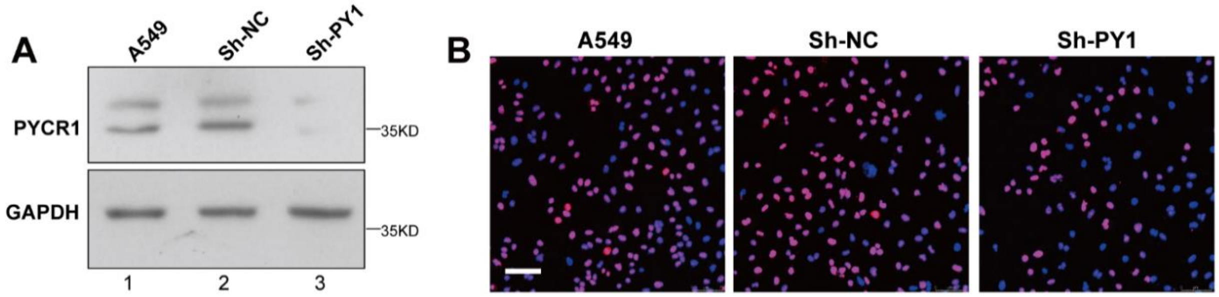

Supplementary Fig.3

Supplementary Figure 3. Knockdown of PYCR1 inhibits cell proliferation

A549 cells were infected with PYCR1 shRNA (Sh-PY1) lentivirus or control lentivirus for 5 days and then analyzed by Western blot with antibodies recognizing PYCR1 and GAPDH (as a loading control (A). Cells (as specified in the figure) were stained with DAPI and anti-Ki67 antibody as described in the Methods (B). Bars,75μm.

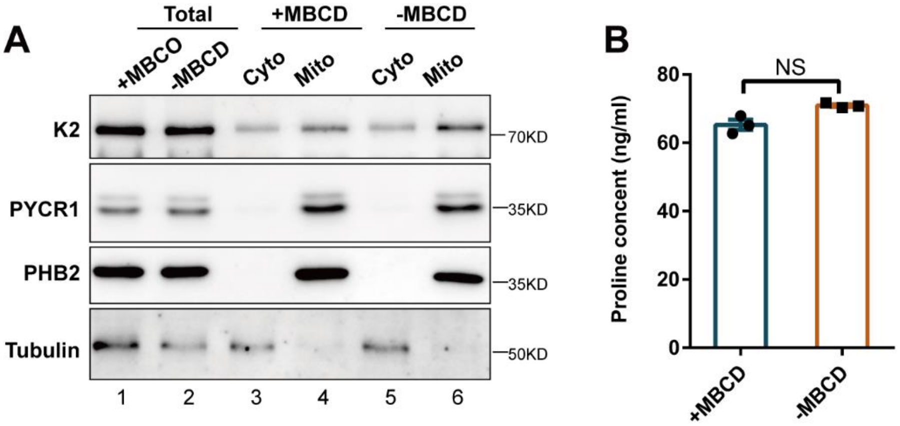

Supplementary Fig.4

Supplementary Figure 4. Suppression of integrin internalization with MBCD does not alter kindlin-2 and PYCR1 localization and proline synthesis

(A) The cytosolic fraction (Cyto, lane 3 and 5), mitochondrial fraction (Mito, lane 4 and 6) and total cell lysates (Total, lane 1 and 3) from A549 cells treated with or without 10mM MBCD on soft substrate were analyzed by Western blotting with antibodies to kindlin-2, PYCR1, tubulin and prohibitin-2 (PHB2) as indicated. (B) A549 on soft substrate were pretreated with or without 10mM MBCD for 1 h, The proline levels in the cells (as specified in the figure) were analyzed as described in the Methods. The results were quantified (B). n=3. NS, no significance.

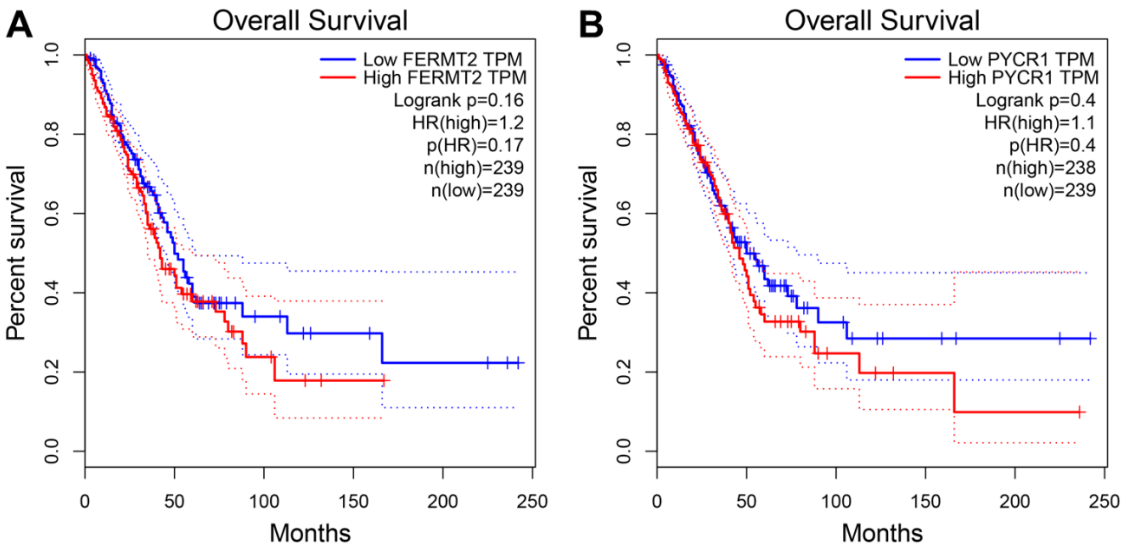

Supplementary Fig.5

Supplementary Figure 5. Overall survival rates of human lung adenocarcinoma patients with high or low mRNA levels of kindlin-2 (FERMT2) and PYCR-1

Survival analysis between the mRNA expression levels of *kindlin-2 (FERMT2)* (A) and *PYCR-1*(B) genes and overall survival time in human lung adenocarcinoma was based on the data from the Gene Expression Profiling Interactive Analysis database (http://gepia.cancer-pku.cn). Red line indicates the samples with gene highly expressed, and blue line shows the samples with gene lowly expressed. HR, hazard ratio. TPM, transcripts per Kilobase million.

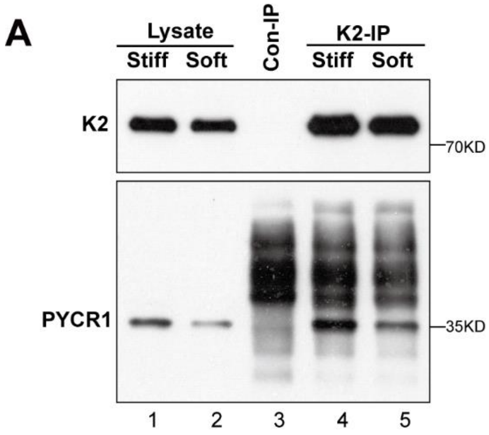

Supplementary Fig.6

Supplementary Figure 6. ECM stiffening promotes the formation of the kindlin-2-PYCR1 complex in mesenchymal stem cells

(A) Human mesenchymal stem cells (MSCs) were plated on soft or stiff collagen-I-coated hydrogels for 48 hours and analyzed by IP with anti-kindlin-2 antibody. The cell lysates (lanes 1 and 2) and IP samples (lanes 4 and 5) were analyzed by Western blotting with antibodies recognizing PYCR1 or kindlin-2. Lane 3, the sample was prepared as that of lane 4 except anti-kindlin-2 antibody was substituted with irrelevant mouse IgG (as a control).

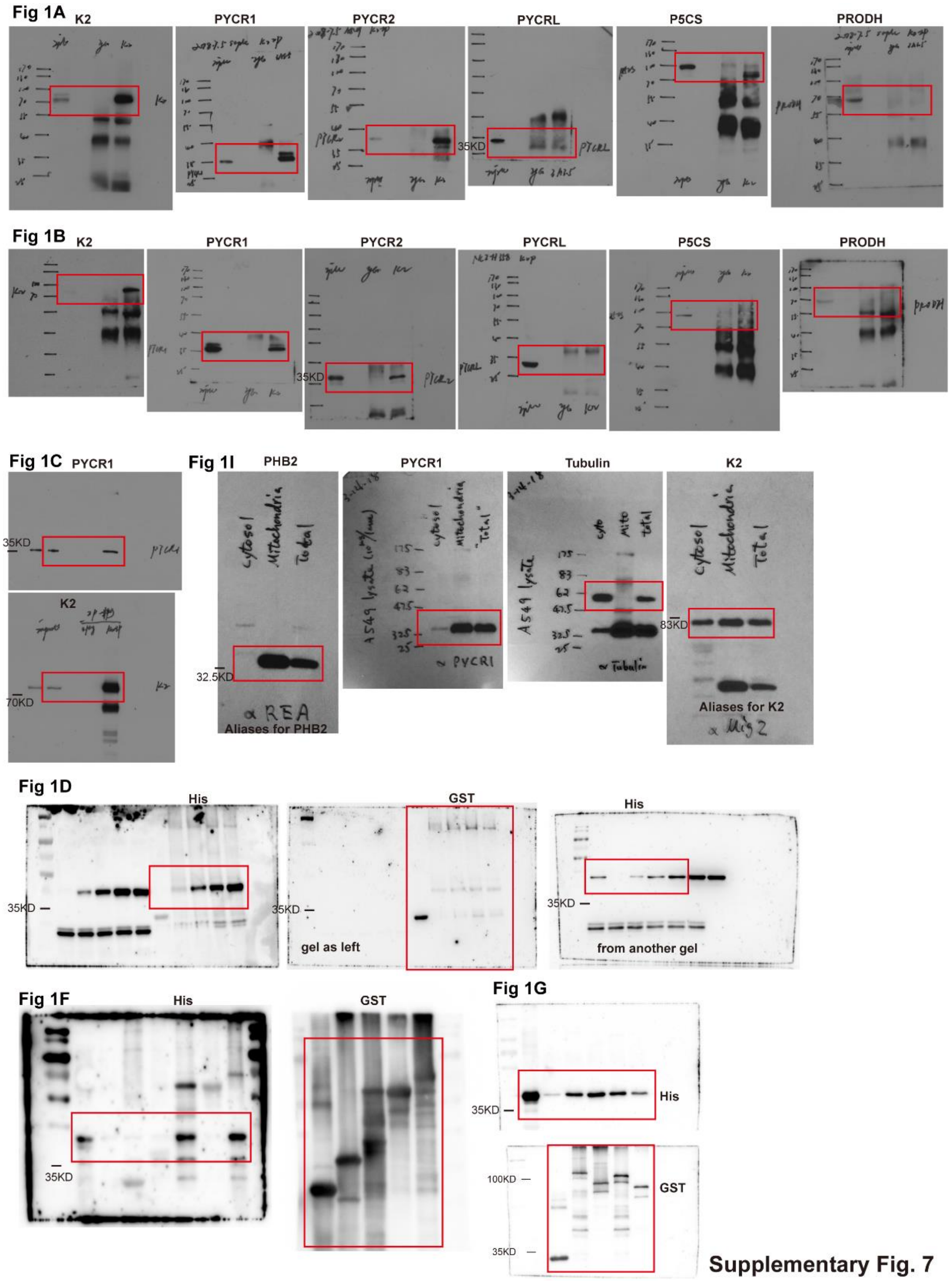

Supplementary Figure 7. Uncropped Western blots for Figure 1

Uncropped Western blots from which the cropped Western blots shown in Figs. 1A, 1B, 1C, 1D, 1F,1G and 1I, were derived are shown. The red boxes outline the approximate areas that were cropped.

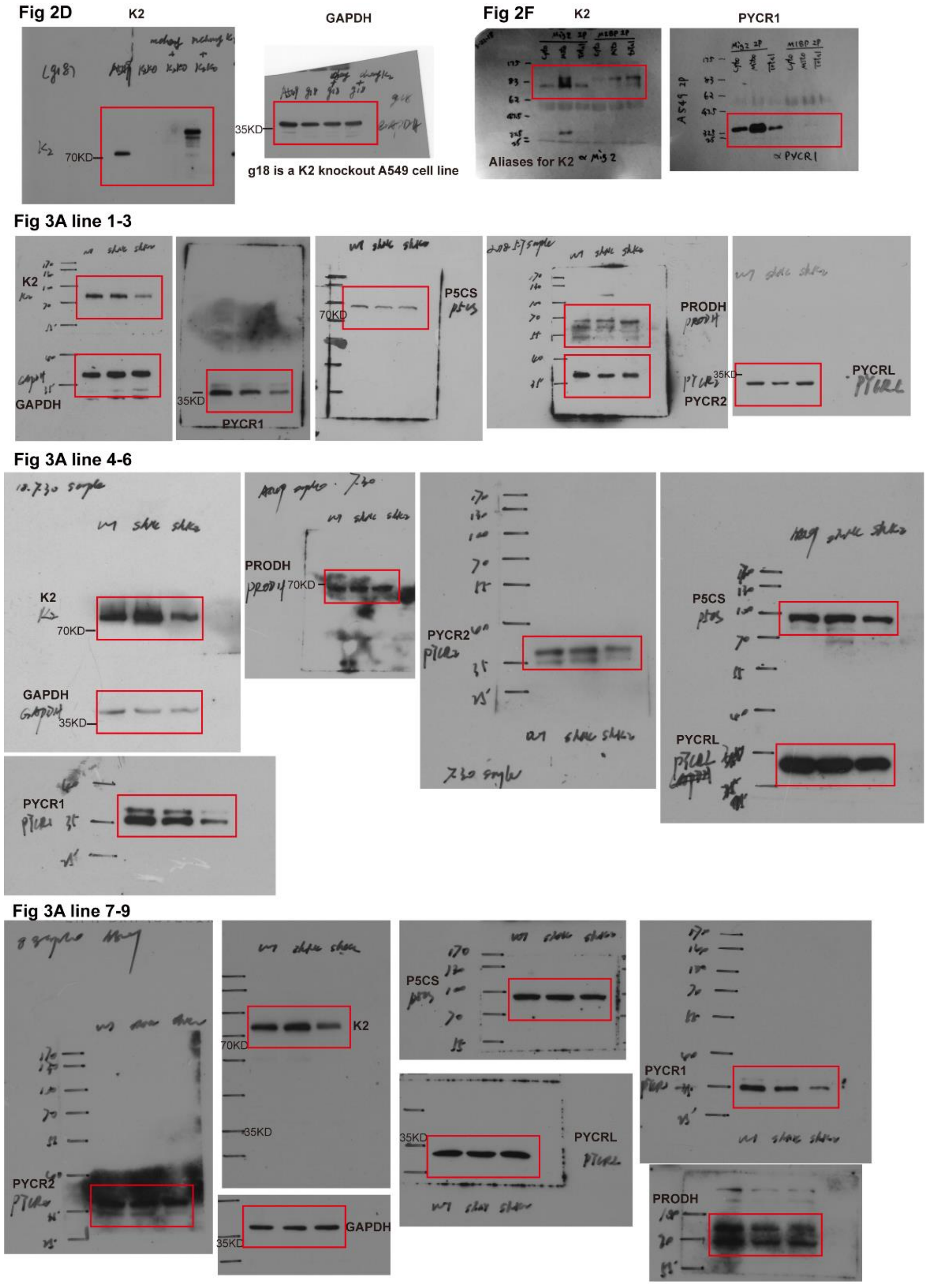

Supplementary Fig. 8

Supplementary Figure 8. Uncropped Western blots for Figures 2 and 3

Uncropped Western blots from which the cropped Western blots shown in Figs. 2D and 3A were derived are shown. The red boxes outline the approximate areas that were cropped.

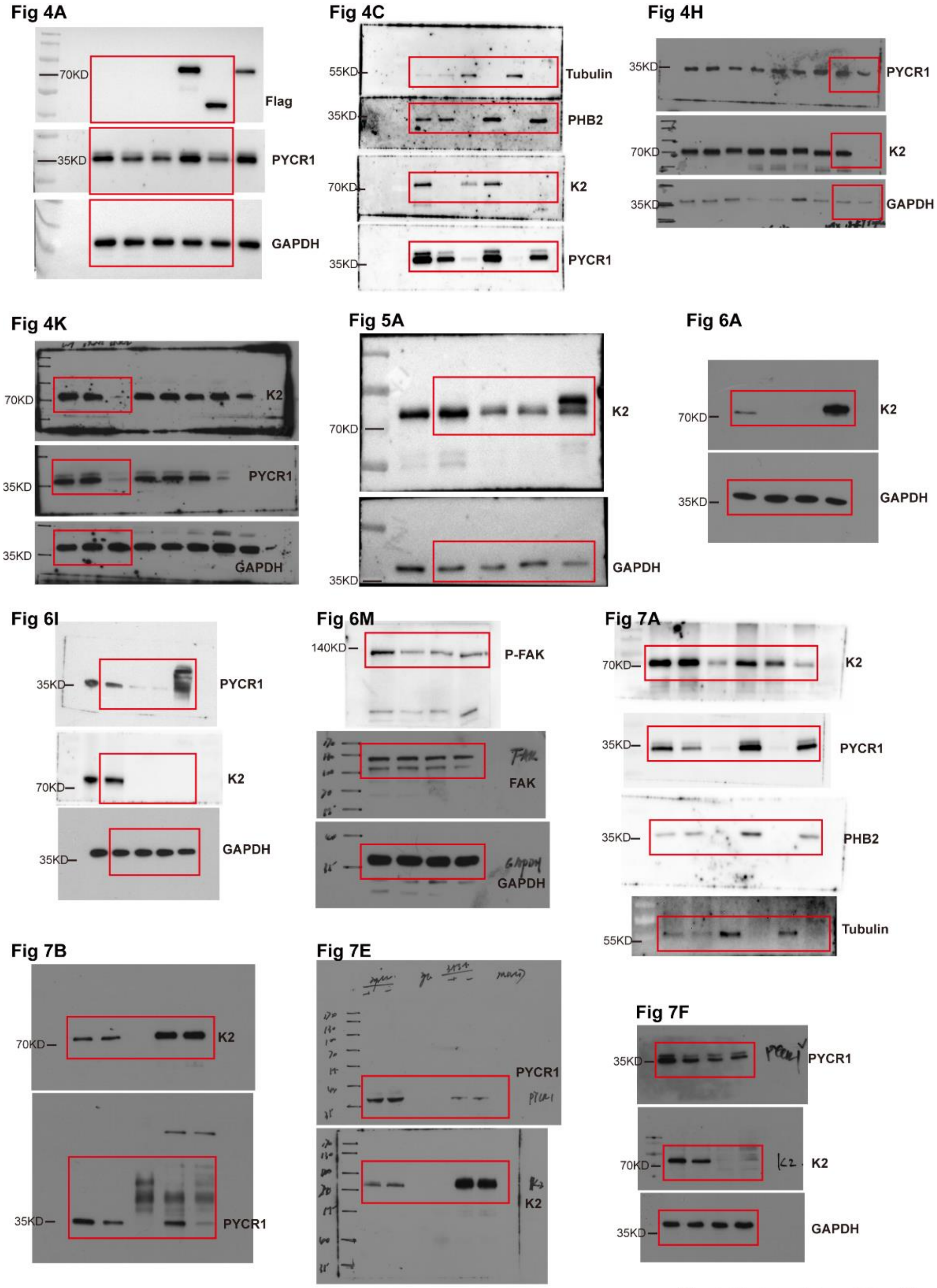

Supplementary Fig. 9

Supplementary Figure 9. Uncropped Western blots for Figures 4-7

Uncropped Western blots from which the cropped immunoblots shown in Figure 4A, 4C, 4G, 4L, 5A, 6A, 6I, 6M, 7A, 7B, 7E and 7F were derived are shown. The red boxes outline the approximate areas that were cropped.

Supplementary Table 1. Clinical information of human lung adenocarcinoma and normal adjacent lung tissues (Lung cancer tissue microarray LC953, Alenabio) used in the immunohistochemical staining analyses

Pos.	No.	Sex	Age	Organ/Anatomic Site	Pathology diagnosis	Grade	Stage	Type	pTNM
C3	27	F	53	Lung	Adenocarcinoma	2	T2N0M0	Malignant	IB
C4	28	F	53	Lung	Adenocarcinoma	2	T2N0M0	Malignant	IB
C5	29	F	65	Lung	Adenocarcinoma	2	T2N2M0	Malignant	IIIA
C6	30	F	65	Lung	Adenocarcinoma	2	T2N2M0	Malignant	IIIA
C7	31	M	42	Lung	Adenocarcinoma	2	T2N0M0	Malignant	IB
C8	32	M	42	Lung	Adenocarcinoma	2	T2N0M0	Malignant	IB
C9	33	F	67	Lung	Adenocarcinoma	2	T2N3M0	Malignant	IIIB
C10	34	F	67	Lung	Adenocarcinoma	2	T2N3M0	Malignant	IIIB
C11	35	F	49	Lung	Adenocarcinoma	2	T2N1M0	Malignant	IIA
C12	36	F	49	Lung	Adenocarcinoma	2	T2N1M0	Malignant	IIA
D1	37	M	47	Lung	Adenocarcinoma	2	T2N1M0	Malignant	II
D2	38	M	47	Lung	Adenocarcinoma	2	T2N1M0	Malignant	II
D3	39	M	73	Lung	Adenocarcinoma	3	T3N1M0	Malignant	IIIA
D4	40	M	73	Lung	Adenocarcinoma	3	T3N1M0	Malignant	IIIA
D5	41	F	30	Lung	Adenocarcinoma	3	T4N1M1	Malignant	IV
D6	42	F	30	Lung	Adenocarcinoma	3	T4N1M1	Malignant	IV
D7	43	F	63	Lung	Adenocarcinoma	3	T2N1M0	Malignant	IIB

D8	44	F	63	Lung	Adenocarcinoma	3	T2N1M0	Malignant	IIB
D9	45	F	48	Lung	Adenocarcinoma	3	T2N0M0	Malignant	IB
D10	46	F	48	Lung	Adenocarcinoma	3	T2N0M0	Malignant	IB
D11	47	M	53	Lung	Adenocarcinoma	3	T2N0M0	Malignant	IB
D12	48	M	53	Lung	Adenocarcinoma	3	T2N0M0	Malignant	IB
E1	49	M	59	Lung	Adenocarcinoma	3	T2N0M0	Malignant	IB
E2	50	M	59	Lung	Adenocarcinoma	3	T2N0M0	Malignant	IB
H1	85	M	42	Lung	Cancer adjacent lung tissue from No.31				
H2	86	M	42	Lung	Cancer adjacent lung tissue from No.32				
H3	87	F	48	Lung	Cancer adjacent lung tissue from No.45				
H4	88	F	48	Lung	Cancer adjacent lung tissue from No.46				
H7	91	F	67	Lung	Cancer adjacent lung tissue from No.33				
H8	92	F	67	Lung	Cancer adjacent lung tissue from No.34				
H9	93	M	53	Lung	Cancer adjacent lung tissue from No.47				
H10	94	M	53	Lung	Cancer adjacent lung tissue from No.48				

Supplementary Table 2. List of Primers Used for Quantitative RT-PCR

Gene	Sequence (5' to 3')	
GAPDH (h)	Sense	CCAGAACATCATCCCTGCCTCTACT
	Antisense	GGTTTTTCTAGACGGCAGGTCAGGT
PYCR1 (h)	Sense	GAAGATGGGGGTGAAGTTGA
	Antisense	CTCAATGGAGCTGATGGTGA
