# Supplementary material for: Kindlin-2 links mechano-environment to proline synthesis and tumor growth
Source: Nat Commun. 2019 Feb 19;10:845. doi: 10.1038/s41467-019-08772-3 (PMC6381112; doi:10.1038/s41467-019-08772-3)
Supplement: Supplementary file 2 — Reporting Summary [file 41467_2019_8772_MOESM2_ESM.pdf]

nature research

Corresponding author(s): Chuanyue Wu and Ling Guo

Last updated by author(s): Dec 31, 2018

Reporting Summary

Nature Research wishes to improve the reproducibility of the work that we publish. This form provides structure for consistency and transparency in reporting. For further information on Nature Research policies, see Authors & Referees and the Editorial Policy Checklist.

Statistics

For all statistical analyses, confirm that the following items are present in the figure legend, table legend, main text, or Methods section.

n/a	Confirmed	
☐	☒	The exact sample size (n) for each experimental group/condition, given as a discrete number and unit of measurement
☐	☒	A statement on whether measurements were taken from distinct samples or whether the same sample was measured repeatedly
☐	☒	The statistical test(s) used AND whether they are one- or two-sided<br>*Only common tests should be described solely by name; describe more complex techniques in the Methods section.*
☒	☐	A description of all covariates tested
☒	☐	A description of any assumptions or corrections, such as tests of normality and adjustment for multiple comparisons
☐	☒	A full description of the statistical parameters including central tendency (e.g. means) or other basic estimates (e.g. regression coefficient) AND variation (e.g. standard deviation) or associated estimates of uncertainty (e.g. confidence intervals)
☒	☐	For null hypothesis testing, the test statistic (e.g. F, t, r) with confidence intervals, effect sizes, degrees of freedom and P value noted<br>*Give P values as exact values whenever suitable.*
☒	☐	For Bayesian analysis, information on the choice of priors and Markov chain Monte Carlo settings
☒	☐	For hierarchical and complex designs, identification of the appropriate level for tests and full reporting of outcomes
☒	☐	Estimates of effect sizes (e.g. Cohen's d, Pearson's r), indicating how they were calculated

Our web collection on statistics for biologists contains articles on many of the points above.

Software and code

Policy information about availability of computer code

Data collection	We have analyzed the mRNA levels of both kindlin-2 (FERMT2) and PYCR-1 using GEPIA (Gene Expression Profiling Interactive Analysis, http://gepia.cancer-pku.cn).
Data analysis	Quantitation of band intensities was performed with Image J; Immunohistochemical staining was quantified using Image-Pro Plus software version 6.

For manuscripts utilizing custom algorithms or software that are central to the research but not yet described in published literature, software must be made available to editors/reviewers. We strongly encourage code deposition in a community repository (e.g. GitHub). See the Nature Research guidelines for submitting code & software for further information.

Data

Policy information about availability of data

All manuscripts must include a data availability statement. This statement should provide the following information, where applicable:

- Accession codes, unique identifiers, or web links for publicly available datasets
- A list of figures that have associated raw data
- A description of any restrictions on data availability

All relevant data are available from the authors. The mass spectrometry data have been deposited in MassIVE with accession code MSV000083291 [ftp://massive.ucsd.edu/MSV000083291].. Uncropped Western blots for Figs. 1A, 1B, 1C, 1D, 1F,1G, 1I, 2D, 3A, 4A, 4C, 4G, 4L, 5A, 6A, 6I, 6M, 7A, 7B, 7E and 7F are provided in Supplementary Figs. 7-9. A reporting summary for this article is available as a Supplementary Information file.

Field-specific reporting

Please select the one below that is the best fit for your research. If you are not sure, read the appropriate sections before making your selection.

☒ Life sciences ☐ Behavioural & social sciences ☐ Ecological, evolutionary & environmental sciences

For a reference copy of the document with all sections, see nature.com/documents/nr-reporting-summary-flat.pdf

Life sciences study design

All studies must disclose on these points even when the disclosure is negative.

Sample size	Sample size was not pre-determined and it is indicated in the Figure legends.
Data exclusions	No data were excluded from the analyses.
Replication	All experiments were repeated at least 3 times, unless stated differently in figure legends. The main observations of the work were reproduced in different human cell lines and mice, in different experimental settings and by different technologies.<br>Sample size and number of independent experiments are clearly stated in the figure legend.
Randomization	No randomization techniques were used.
Blinding	Investigators were not blinded to group allocation during data collection and analysis.

Reporting for specific materials, systems and methods

We require information from authors about some types of materials, experimental systems and methods used in many studies. Here, indicate whether each material, system or method listed is relevant to your study. If you are not sure if a list item applies to your research, read the appropriate section before selecting a response.

Materials & experimental systems

n/a	Involved in the study	
☐	☒	Antibodies
☐	☒	Eukaryotic cell lines
☒	☐	Palaeontology
☐	☒	Animals and other organisms
☒	☐	Human research participants
☒	☐	Clinical data

Methods

n/a	Involved in the study	
☒	☐	ChIP-seq
☒	☐	Flow cytometry
☒	☐	MRI-based neuroimaging

Antibodies

Antibodies used	We described all antibodies that we used, including supplier name, catalog number and dilutions in the methods.
Validation	Validations are based on the datasheets from the manufactures.

Eukaryotic cell lines

Policy information about cell lines

Cell line source(s)	ATCC
Authentication	Cell-lines were authenticated by ATCC.
Mycoplasma contamination	Prior to shipping each cell line, the ATCC performed mycoplasma testing.
Commonly misidentified lines<br>(See ICLAC register)	A549 and NCI-H358 that we used are not in the list of misidentified cell lines.

Animals and other organisms

Policy information about studies involving animals; ARRIVE guidelines recommended for reporting animal research

Laboratory animals	Age-matched mice (6-8 weeks old) of both sexes (as specified in each experiment) were used.

Wild animals	The study did not involve wild animals.
Field-collected samples	The study did not involve samples collected from the field.
Ethics oversight	All mouse work was performed with the approval of the Institutional Animal Care and Use Committee, Southern University of Science and Technology.

Note that full information on the approval of the study protocol must also be provided in the manuscript.
